# Escape from Lethal Bacterial Competition through Coupled Activation of Antibiotic Resistance and a Mobilized Subpopulation

**DOI:** 10.1371/journal.pgen.1005722

**Published:** 2015-12-08

**Authors:** Reed M. Stubbendieck, Paul D. Straight

**Affiliations:** 1 Interdisciplinary Program in Genetics, Texas A&M University, College Station, Texas, United States of America; 2 Department of Biochemistry and Biophysics, Texas A&M University, College Station, Texas, United States of America; A*STAR, SINGAPORE

## Abstract

Bacteria have diverse mechanisms for competition that include biosynthesis of extracellular enzymes and antibiotic metabolites, as well as changes in community physiology, such as biofilm formation or motility. Considered collectively, networks of competitive functions for any organism determine success or failure in competition. How bacteria integrate different mechanisms to optimize competitive fitness is not well studied. Here we study a model competitive interaction between two soil bacteria: *Bacillus subtilis* and *Streptomyces* sp. Mg1 (*S*. Mg1). On an agar surface, colonies of *B*. *subtilis* suffer cellular lysis and progressive degradation caused by *S*. Mg1 cultured at a distance. We identify the lytic and degradative activity (LDA) as linearmycins, which are produced by *S*. Mg1 and are sufficient to cause lysis of *B*. *subtilis*. We obtained *B*. *subtilis* mutants spontaneously resistant to LDA (LDA^R^) that have visibly distinctive morphology and spread across the agar surface. Every LDA^R^ mutant identified had a missense mutation in *yfiJK*, which encodes a previously uncharacterized two-component signaling system. We confirmed that gain-of-function alleles in *yfiJK* cause a combination of LDA^R^, changes in colony morphology, and motility. Downstream of *yfiJK* are the *yfiLMN* genes, which encode an ATP-binding cassette transporter. We show that *yfiLMN* genes are necessary for LDA resistance. The developmental phenotypes of LDA^R^ mutants are genetically separable from LDA resistance, suggesting that the two competitive functions are distinct, but regulated by a single two-component system. Our findings suggest that a subpopulation of *B*. *subtilis* activate an array of defensive responses to counter lytic stress imposed by competition. Coordinated regulation of development and antibiotic resistance is a streamlined mechanism to promote competitive fitness of bacteria.

## Author Summary

Antibiotics are one mechanism among many that bacteria use to compete with each other. Bacteria in the environment and in host organisms likely use networks of competitive mechanisms to survive and to shape the composition and function of diverse communities. In this study, we cultured two species of soil bacteria to observe the outcome of competition and to identify competitive functions that dictate the outcome. We show that one organism, *Streptomyces* sp. Mg1, produces antibiotic linearmycins that cause cellular lysis and degradation of a competing colony of *Bacillus subtilis*. In turn, the *B*. *subtilis* activate a resistance mechanism, either transiently or through mutation of a two-component signaling system. Activation of the signaling system produces a suite of identified responses, which include resistance to linearmycins, altered colony morphology that resembles biofilms, and enhanced motility of *B*. *subtilis*. This work identifies a unified, multifaceted survival response that is induced by a subpopulation of bacteria to escape lethal consequences of antibiotic-mediated competition.

## Introduction

Bacteria are communal organisms. As such, bacteria have mechanisms to interact with other species that range from cooperative to antagonistic. Antibiotics are a classic example of molecules produced by bacteria that probably function in shaping microbial communities due to their bioactive function, including growth inhibitory and stimulatory activities [1–4]. The study of antibiotics has revealed a great deal about the cellular functions they target, mechanisms of resistance, and uses in treating disease. The traditional approach to discovery of antibiotics typically begins with extraction of metabolites from culture media, followed by direct screening of culture extracts to identify growth inhibitory agents [5]. While this approach has had tremendous success for antibiotic discovery, it has left great gaps in our understanding of competitive dynamics between bacteria. Approaches to bacterial competition that rely on culture of two or more organisms together are emerging as a powerful tool to discover new bioactive molecules and reimagine mechanisms of competition between diverse species of bacteria [6,7]. For instance, microbial competitive functions include secreted enzymes, type VI secretion systems, and specialized metabolism, including developmental signals and antibiotics [1,4,8]. In addition, changes in community functions such as biofilm formation or motility are recognized increasingly as important competitive strategies for bacteria [9,10].

Specialized metabolism and developmental functions are common features among soil bacteria, including the actinomycetes, bacilli, and myxobacteria [11–17]. In these bacteria, antibiotic production and cellular development are often intertwined and co-regulated processes, which is thought to provide fitness benefits to the organisms [18–20]. For example, during typical development *Streptomyces* species differentiate and develop spores [14]. During *Streptomyces* sporulation the substrate mycelium is cannibalized, which is thought to provide the cells with necessary nutrients to complete sporulation [21,22]. Cannibalization of the substrate mycelium is concurrent with production of many antibiotics, which are thought to protect the nutrient resources from opportunistic competitors [23]. Use of simple, tractable assays of two or more competing bacteria is one approach to identify new specialized metabolites, enzymes, and bacterial functions that determine the outcomes of competitive interactions. Indeed, interaction assays reveal not only growth inhibitory metabolites, but also changes in development and colony morphology that expose abundant and poorly understood survival mechanisms for bacteria. Dynamic patterns of interaction based on models of competition are producing new insights into bacterial competitive mechanisms [9,18,24–28].

As a model for competitive interactions, we use different species of *Bacillus* and *Streptomyces*. This competition model has led to identification of new functions for known molecules, including bacillaene and surfactin [18,24]. In the case of surfactin, a secreted hydrolase was identified from *Streptomyces* sp. Mg1 (*S*. Mg1) and shown to be a resistance mechanism that specifically degrades surfactin and plipastatin produced by *Bacillus subtilis* [25]. The current study stems from observing colonies of *S*. Mg1 and *B*. *subtilis* placed side by side on agar media. In this format, cellular lysis occurs along with progressive degradation of the *B*. *subtilis* colony [29]. Previously, imaging mass spectrometry revealed the loss of the polyglutamate component of colony extracellular matrix in the area of lysis, indicating degradation of both cellular and extracellular materials [30,31]. *Streptomyces* sp. Mg1 encodes production of many specialized metabolites with potential to participate in lysis and degradation [32]. One gene cluster encodes the biosynthetic enzymes for chalcomycin A, which inhibits the growth of *B*. *subtilis* but does not cause lysis and colony degradation [29]. Here we report both the identification of a lytic degradative activity (LDA) from *S*. Mg1, as well as a mechanism of resistance to LDA for *B*. *subtilis*. We show that resistant mutants of *B*. *subtilis* have a complex phenotype, which includes LDA resistance and visible changes in colony morphology and motility. We show the LDA resistance and the changes in colony morphology and motility are genetically separable functions, all regulated by a two-component system of previously unknown function. Our results indicate that a subpopulation of *B subtilis* cells in a colony trigger a complex mechanism for competitive fitness when challenged by the streptomycete.

## Results

### Identification of the molecule responsible for LDA

**Fig 1:**
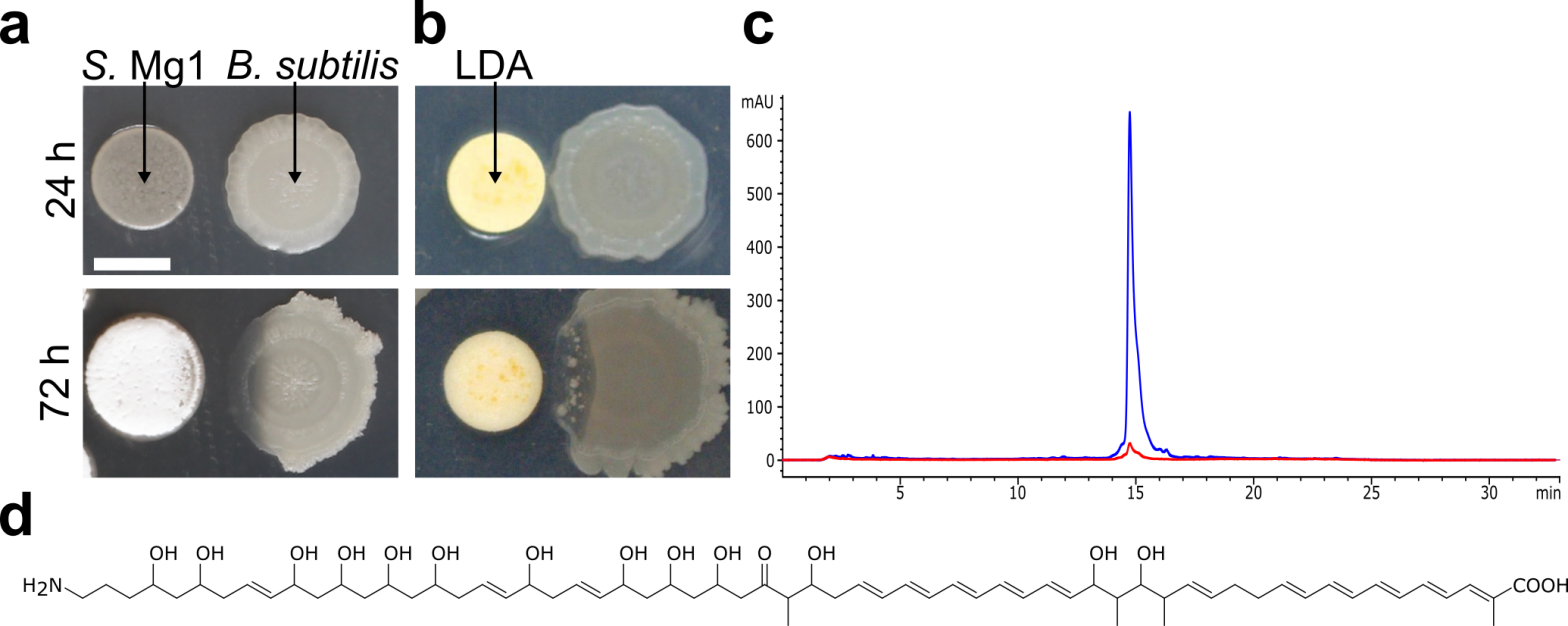
Identification of linearmycin B as the causative agent of LDA. (a) When co-cultured on MYM agar, *S*. Mg1 (left) releases molecule(s) that cause cellular lysis and colony degradation of *B*. *subtilis* (PDS0067) (right) at a distance. (b) We cultured *B*. *subtilis* (PDS0067) (right) alone on MYM7 agar for 24 h before adding isolated LDA onto a filter paper disc (left) adjacent to the colony, which subsequently lysed over 48 h similarly to co-culture with *S*. Mg1. (c) HPLC trace of the isolated LDA. The peak is detected by UV absorbance at 333 nm (blue). The background is shown by the 254 nm absorbing trace (red). (d) The structure of linearmycin B. Scale bar is 5 mm.

When cultured next to *S*. Mg1, *Bacillus subtilis* colonies are progressively degraded and the underlying cells are lysed (Fig 1A) [29]. Progressive degradation of the cells and the extracellular matrix is visible as a translucent patch that develops on a formerly opaque colony (Fig 1A, S1 Movie). Our initial interest was to identify causes of lysis and colony degradation. To identify candidate lytic agents, we chose a direct approach to isolate *S*. Mg1 metabolites or enzymes that contribute to the lytic and degradative activity (LDA). Initially, we found active material present in whole plate butanol extracts from *S*. Mg1 grown on agar. To improve yields and decrease complexity of LDA extracts, we cultured *S*. Mg1 in liquid medium in the presence of non-polar HP-20 resin for adsorption of metabolites. Adsorbed metabolites were eluted using methanol to generate the crude extract. *Bacillus subtilis* colonies exposed to the crude extract lysed, indicating the presence of the activity. To isolate the active agent, we fractionated the crude extract, first using a stepwise (10%) methanol gradient followed by time-based HPLC fractionation, and tested for active fractions (see methods for a detailed description). The *Δpks* strain of *B*. *subtilis* was used for enhanced sensitivity in these assays, because the mutant is hypersensitive to lysis in co-culture with *S*. Mg1 [29]. We isolated a single peak from a HPLC fraction that caused lysis and colony degradation similar to *S*. Mg1 (Fig 1A and 1C). The similarity between the effects of isolated LDA and a competing *S*. Mg1 colony suggested that lysis and colony degradation of *B*. *subtilis* may result from the action of a single compound.

To identify the active molecule, we analyzed the HPLC-purified sample by UV absorbance and ESI-mass spectrometry. The molecule showed strong UV absorbances at 319, 333, and 351 nm, indicative of a conjugated pentaene moiety [33]. We found that the observed mass, 1166.7 [M+H]^+^ (S1A Fig) and the UV absorbance profile are consistent with linearmycin B (Fig 1D), a linear polyene antibiotic produced by *Streptomyces* sp. no. 30 [34,35]. We used 1D and 2D NMR of the active sample to confirm the presence of linearmycin B. Previously reported chemical shifts for linearmycin B accorded with those we obtained for the LDA sample (S1B Fig, S1 Table) [35]. The linearmycins were originally identified as a pair of compounds, linearmycin A and B [34,35]. We examined the crude extracts and found them to also contain linearmycin A, which is also active for lysis of *B*. *subtilis* (*m/z* 1140) (S2 Fig). Furthermore, the *S*. Mg1 genome (GenBank Accession CP011664) [32] includes a polyketide gene cluster predicted to be responsible for linearmycin biosynthesis. We tested a mutant strain, *S*. Mg1-*Δ37*, which contains a chromosome truncation that removes the linearmycin biosynthetic cluster, and found the mutant failed to lyse *B*. *subtilis* or produce linearmycins (S2 Fig). In a parallel study, a targeted deletion of the acyl-transferase encoding gene in the linearmycin biosynthetic gene cluster disrupts linearmycin production specifically and blocks all lytic activity from the strain (personal communication, B. Chris Hoefler). Taken together, we conclude that *S*. Mg1 produces linearmycins, which are sufficient for LDA against *B*. *subtilis*. For simplicity, we collectively refer to these molecules as LDA.

No mechanism is known for either growth inhibition or the lytic effect that we observe with LDA. Linearmycin A was originally shown to inhibit growth of *Escherichia coli* and *Staphylococcus aureus* in addition to three fungal species, but no antibacterial mechanism of action was reported [35]. Structurally related polyene antibiotics include antifungal agents such as amphotericin B [36], nystatin [37], and ECO-02301 [38]. Amphotericin B and nystatin inhibit fungal growth specifically by interactions with ergosterol and the fungal plasma membrane [39–42]. However, bacterial membranes lack ergosterol, suggesting a different mechanism of action against bacteria for LDA. Nystatin was found to induce biofilm formation by *B*. *subtilis* grown in LB media [43], demonstrating that antifungal polyenes are biologically active in the absence of ergosterol. The lytic activity of LDA indicates a mechanism of action for the linearmycins that differs from nystatin. In the absence of a known target, we sought an approach to better understand the lysis and degradation of *B*. *subtilis*.

### LDA resistance caused by activation of the YfiJK two-component system

**Fig 2:**
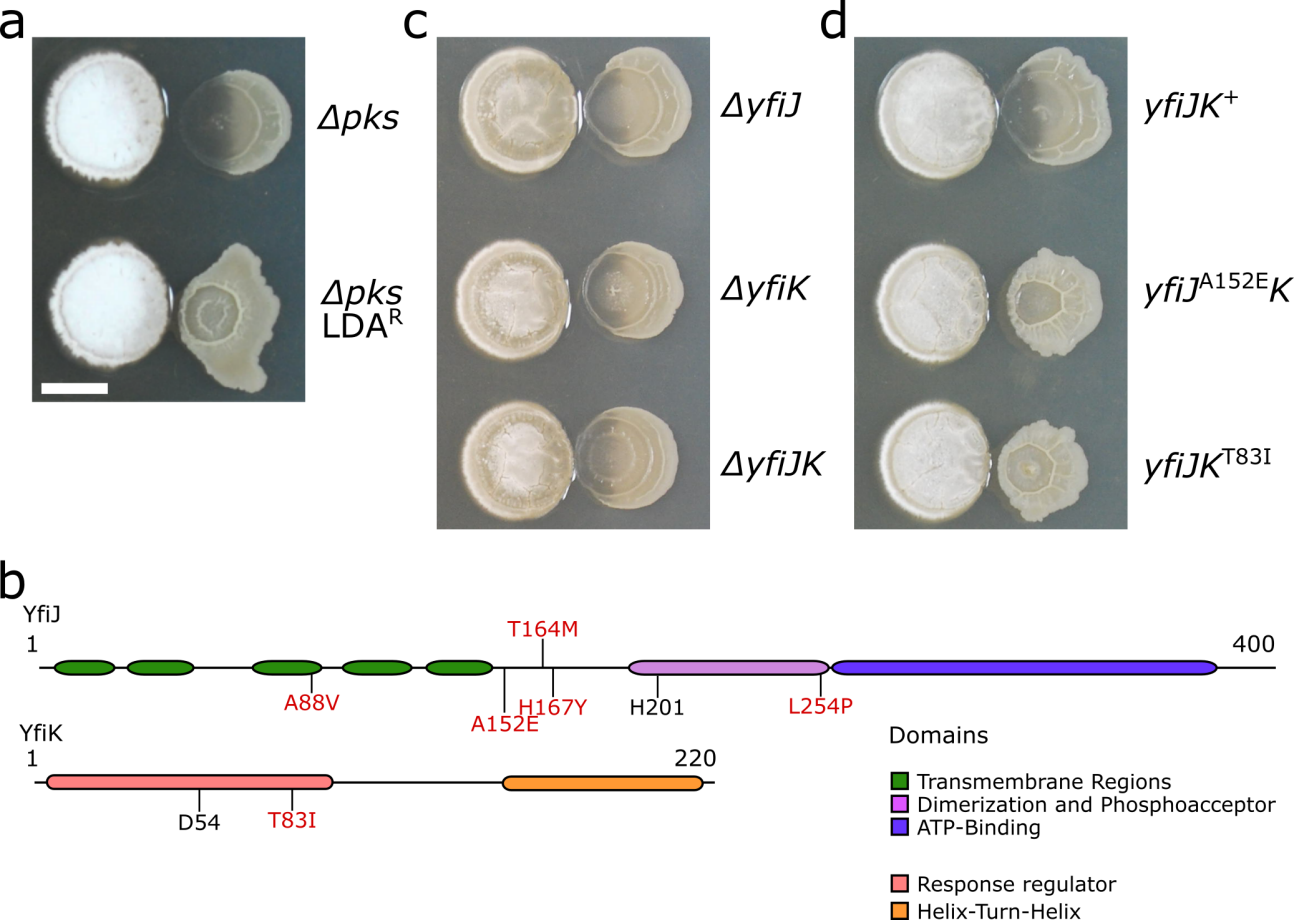
Point mutations in yfiJK are responsible for LDA resistance. (a) The parental strain of *B*. *subtilis*, *Δpks* (PDS0067, top), and a representative spontaneous LDA^R^ mutant (bottom). The colony formed by the parental strain is lysed but the mutant colony remains intact. The spontaneous LDA resistant mutant has a distorted shape and more wrinkled surface than its parental strain. (b) Diagrams of YfiJ and YfiK. The amino acid substitutions identified in spontaneous LDA^R^ mutants are shown in red. The predicted conserved phosphoacceptor residues are shown in black. (c) Strains of *B*. *subtilis* deleted for *yfiJ* (PDS0555) or *yfiK* (PDS0556) independently or *yfiJK* together (PDS0554) are lysed in co-culture with *S*. Mg1. (d) We complemented the *yfiJK* deletion strain by inserting at the non-essential *amyE* locus either wild-type *yfiJK* (PDS0627) or alleles identified in spontaneous resistant strains: *yfiJ* ^A152E^ *K* (PDS0685) and *yfiJK* ^T83I^ (PDS0628). In co-culture with *S*. Mg1, wild-type *yfiJK* ^+^ complement was lysed and degraded, but the strains complemented with LDA^R^ alleles of *yfiJK* were LDA resistant. The complementation strains reproducibly had a more wrinkled morphology than wild type, similar to the spontaneous LDA^R^ strain. All cultures place *S*. Mg1 on the left and *B*. *subtilis* on the right. Photographs were taken after 72 h co-incubation on MYM agar. Scale bar is 5 mm.

When plated next to extracts of LDA or *S*. Mg1 colonies, small *B*. *subtilis* colonies emerge in the region of lysis and appear to be resistant to LDA [29]. We wanted to identify mechanisms of resistance as an approach to better understand the lytic process caused by LDA [44]. Direct comparison of Δ*pks* and wild-type strains of *B*. *subtilis*, either in culture with *S*. Mg1 or when treated with LDA, showed that the Δ*pks* strain is hypersensitive to lysis but has no other observable phenotype in these assays [29]. Therefore as before, we used the Δ*pks* strain of *B*. *subtilis* for these assays, because the LDA hypersensitivity provided an expanded area of lysis in which we could scan for potential resistant mutants. We challenged colonies of the *Δpks* strain of *B*. *subtilis* with extracts from *S*. Mg1 cultures and observed small colonies appearing in the degraded portion of the parent colony after lysis occurred (e.g. Fig 1B). We isolated 60 small colonies from several lysed colonies and tested them for resistance to LDA in co-culture with *S*. Mg1. The majority of the isolates lysed when cultured again with *S*. Mg1, indicating only transient resistance to LDA. However, ten isolates were stably resistant to LDA (LDA^R^), potentially having acquired mutations leading to resistance (Fig 2A). Notably, all of the stable LDA resistant colonies developed a rough, wrinkled colony morphology that is distinct from the parental strain (Fig 2A). Due to a biofilm-like appearance of the LDA^R^ colonies, we suspect the mutations have pleiotropic effects on growth mode and development, as well as resistance to LDA.

To identify the mutant alleles in the LDA^R^ isolates, we sequenced six of the ten mutant genomes and compared the sequences to the parental genome (*Δpks* strain). Surprisingly, all six isolates had point mutations in either of the two genes in the *yfiJK* operon. In addition, eleven non-overlapping mutations occurred in a subset of the spontaneous LDA^R^ mutants (S2 Table). Three spontaneous LDA^R^ mutants possessed point mutations only in *yfiJ*, which prompted our focus on the *yfiJK* operon. Using PCR and Sanger sequencing we found that the other four LDA^R^ isolates also contained point mutations in *yfiJ*. In total, nine of ten mutations were found in *yfiJ* and one in *yfiK* (Fig 2B, Table 1). The *yfiJ* gene encodes a membrane-bound sensor histidine kinase (HK), and the *yfiK* gene encodes its cognate cytoplasmic response regulator (RR) [45]. Together these proteins comprise a two-component system (TCS). In a canonical TCS, a HK dimer senses a signal and autophosphorylates on a conserved histidine residue [46]. The phosphate is subsequently transferred to the cognate RR, which then effects a response, most commonly through DNA binding and regulation of gene expression [46]. In the case of YfiK, the effector domain is a helix-turn-helix domain that likely binds DNA to modulate changes in gene expression [45]. A role in LDA resistance is the first indication of a native function for this two-component system.

**Table 1:** Alleles of *yfiJK* identified in spontaneous LDA^R^ mutants.

Allele	Nucleotide Change	Number Isolated	LDA Resistance
*yfiJ* ^+^	n/a	n/a	–
*yfiJ* ^A88V^	C257T	1	+
*yfiJ* ^A152E^	C455A	1	+
*yfiJ* ^T164M^	C491T	1	+
*yfiJ* ^H167Y^	C499T	6*	+
*yfiJ* ^L254P^	T761C	1^†^	+
*yfiJK* ^+^	n/a	n/a	–
*yfiJK* ^T83I^	C248T	1	+

Each allele is designated by the amino acid substitution. All numbering is with respect to the first amino acid or the first nucleotide of the start codon. Wild-type alleles are included to indicate LDA sensitivity and designated with a superscript ^+^ symbol. The–symbol indicates LDA sensitivity, and the + symbol indicates LDA resistance in co-culture with *S*. Mg1.

*We isolated C499T from three independent experiments.

^†^This resistant mutant was isolated from a transposon-mutagenized strain (see S1 Methods).

To determine whether LDA resistance requires active YfiJK, we deleted *yfiJ* and *yfiK* independently, or *yfiJK* together, in otherwise wild-type genetic backgrounds, and co-cultured these mutants with *S*. Mg1. In all three cases the mutants lysed and were indistinguishable from wild-type *B*. *subtilis* (Fig 2C). The absence of any observable phenotype for the *yfiJK* deletion mutations suggested that resistance arises from gain-of-function alleles that activate the two-component system. As a test for gain-of-function alleles, we genetically complemented the deletion strains of *yfiJ* or *yfiJK* with PCR-amplified alleles from the spontaneous LDA^R^ strains. Control strains complemented with native alleles were wild type with respect to lysis and colony morphology (Fig 2D). Conversely, complementation with the mutant alleles caused *B*. *subtilis* to be resistant to LDA when cultured with *S*. Mg1, and the mutants developed a more wrinkled colony surface than wild type (Fig 2D, Table 1). Based on these observations, we concluded that each LDA^R^ allele is likely activating YfiJK to stimulate both abnormal colony development and LDA resistance.

We next investigated how YfiJK may relate to the mechanism of lysis and colony degradation. We considered the results of a previous microarray study to define the regulon of each known RR in *B*. *subtilis* [47]. In that study, overexpression of *yfiK* repressed expression (≥ 4-fold) of 29 different genes, the majority involved in amino acid biosynthesis [47]. The reported regulon also includes *skfF*, which encodes the ATP-binding cassette (ABC) transporter necessary for release of spore-killing factor (SKF), and *iseA*, a cell wall-associated protein that inhibits two major autolysins [48–50]. We hypothesized that SKF and autolysis might be involved in linearmycin-induced lysis, and that *yfiJK* may regulate the expression of those functions. We tested sensitivity to LDA using four strains of *B*. *subtilis*. First, we tested a strain unable to produce SKF (*ΔskfA-H*) to determine if the cannibalism peptide functions as a lytic agent. Second, we tested whether a strain deficient in *iseA* would show enhanced lysis in the absence of an autolysin inhibitor. Third, because *iseA* regulates autolysins, we tested whether a strain deficient in production of three major autolysins (*ΔlytABC ΔlytD ΔlytF*) may show diminished lysis when exposed to LDA. Fourth, we tested a strain with a deletion of the major motility/autolysin regulator (*ΔsigD*) [51]. All four strains lysed when cultured with *S*. Mg1, indicating that SKF and autolysis do not likely contribute to the lysis mechanism (S3 Fig). In a parallel approach, we used transposon mutagenesis to identify genes in *B*. *subtilis* that may cause lysis under linearmycin-induced stress. We obtained a single, stable LDA^R^ mutant, however LDA resistance was unlinked to the site of transposon insertion in this strain. We sequenced the mutant genome and identified an additional point mutation in *yfiJ* (*yfiJ* ^L254P^) (Table 1, S1 Methods). Thus, using multiple approaches to identify functions conferring LDA resistance, we have found only apparent gain-of-function alleles in *yfiJK*.

### LDA resistance requires YfiJK with active phosphotransfer function

Two-component signaling systems require conserved phosphoacceptor residues for activation and downstream signaling [46]. We identified the phosphoacceptor histidine (H201) in YfiJ and the phosphoacceptor aspartate (D54) in YfiK using multiple sequence alignment to experimentally characterized TCS. Using site-directed mutagenesis we disrupted the phosphoacceptor residues and created the new alleles *yfiJ* ^H201N^ and *yfiK* ^D54A^. As anticipated based on the Δ*yfiJK* phenotype, both phosphoacceptor mutants were sensitive to LDA when cultured with *S*. Mg1 (Table 2). Next, we constructed new alleles that combined the phosphoacceptor disruptions with substitutions found in LDA^R^ alleles to generate the new, double mutant alleles *yfiJ* ^A152E, H201N^ and *yfiK* ^D54A, T83I^. When cultured with *S*. Mg1 these mutant strains lysed, which confirmed the disruption of the gain-of-function LDA^R^ phenotype in the absence of functional a TCS (Table 2). As a final test that LDA resistance results from specific downstream signaling of YfiJK, we constructed a pair of double mutants: (*i*) combining the LDA^R^ allele *yfiJ* ^A152E^ with the phosphoacceptor disruption *yfiK* ^D54A^ and (*ii*) combining the phosphoacceptor disruption *yfiJ* ^H201N^ with the LDA^R^ allele *yfiK* ^T83I^. When these strains were cultured with *S*. Mg1 they were sensitive to LDA (Table 2). These results suggest that LDA resistance is due to specific downstream signaling of YfiJK, leading us to conclude that LDA resistance is due specifically to activation of the TCS.

**Table 2:** LDA resistance requires phosphoacceptor residues.

Allele	LDA Resistance
*yfiJ* ^A152E^	+
*yfiJ* ^H201N^	−
*yfiJ* ^A152E, H201N^	−
*yfiJK* ^T83I^	+
*yfiJK* ^D54A^	−
*yfiJK* ^D54A, T83I^	−
*yfiJ* ^A152E^ *K* ^D54A^	−
*yfiJ* ^H201N^ *K* ^T83I^	−

The conserved phosphoacceptor residues in YfiJ (H201) and YfiK (D54) were mutated to non-phosphorylatable residues. The + symbol indicates LDA resistance in co-culture with *S*. Mg1. The–symbol indicates LDA sensitivity. All numbering is relative to the first amino acid of the YfiJ and YfiK proteins.

### LDA^R^ alleles show specificity for linear polyene metabolites

**Fig 3:**
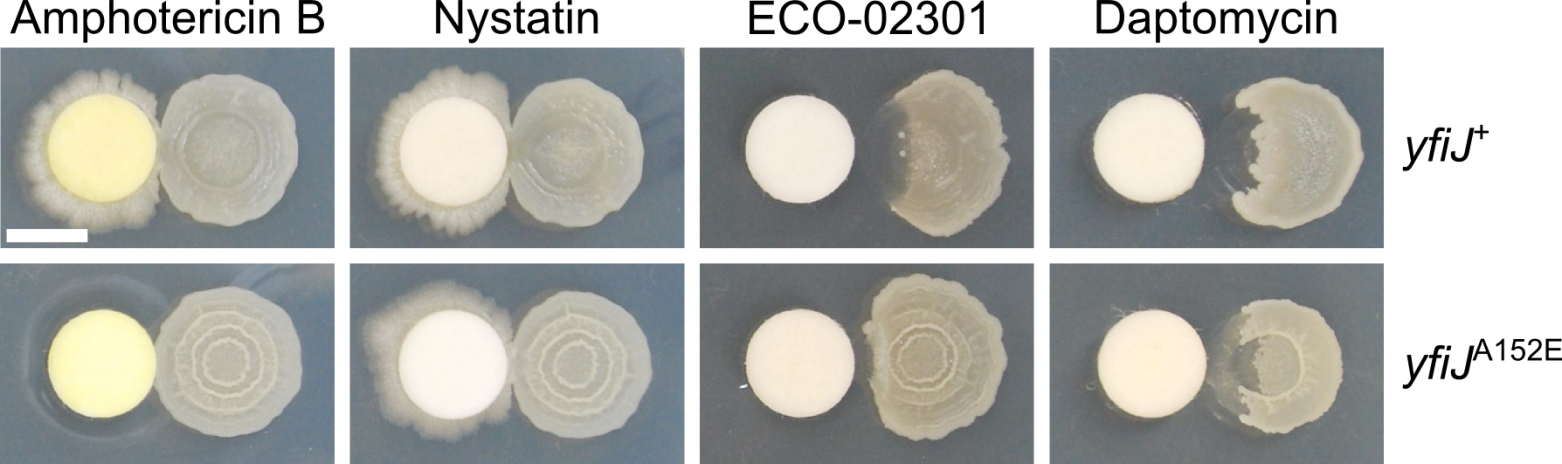
LDA^R^ alleles are specific to LDA caused by linear polyenes. Strains of *B*. *subtilis* (right) were pre-incubated for 24 h on MYM agar before exposure to the antibiotics on filter paper discs (left). Colonies were photographed 48 hours after antibiotic exposure. Amphotericin B (500 μg/filter disc) and nystatin (500 μg/filter disc) are not lytic to *B*. *subtilis*, which can grow around the filter paper disc. A *yfiJ* ^+^ strain of *B*. *subtilis* (PDS0571) is lysed by the linear polyene ECO-02301 (6.25 μg/filter disc) but a strain with the LDA^R^ allele *yfiJ* ^A152E^ (PDS0572) shows resistance to ECO-02301 at this concentration. However both *yfiJ* ^*+*^ and *yfiJ* ^A152E^ strains are susceptible to daptomycin-induced lysis (250 μg/filter disk). Scale bar is 5 mm.

Amphotericin B and nystatin are cyclic polyene antifungals [36,37]. The structurally related linear polyene, linearmycin A, is also antifungal but has also been shown to have antibacterial activity as well [35]. We tested amphotericin B, nystatin, and ECO-02301, a polyene structurally related to the linearmycins [38], for activity against *B*. *subtilis*. ECO-02301 caused lysis similar to linearmycins, but the macrocyclic polyenes amphotericin B and nystatin were not lytic (Fig 3). When tested against purified ECO-02301, the LDA^R^ mutant (*yfiJ* ^A152E^) strain appeared to be partially resistant in this assay (Fig 3). We next sought a quantitative measure of the difference between LDA resistance and sensitivity to LDA and ECO-02301. First, we measured the minimum lytic concentration (MLC) for ECO-02301 using a quantitative agar diffusion assay and determined that a LDA^R^ strain of *B*. *subtilis* was 3.65-fold more resistant to ECO-02301 (Table 3). We applied the same assay to LDA, containing both linearmycin A and B, isolated from *S*. Mg1 cultures and quantified the fold difference in resistance between LDA^R^ and wild-type *B*. *subtilis*. The LDA^R^ mutant was nearly ten-fold more resistant to LDA compared to the sensitive strain (Table 3). The difference in relative resistance to ECO-02301 and LDA may be in part due to structural differences in the molecules. The synthesis of ECO-02301 includes tailoring reactions that glycosylate the polyketide backbone and condense an amidohydroxycyclopentenone moiety onto the terminal carboxylic acid group [38,52]. The structural differences may affect target affinity, solubility, or other properties of the molecule, leading to differences in overall activity.

**Table 3:** Minimum lytic concentrations.

Antibiotic	*yfiJ* ^+^ MLC (μg/mL)	*yfiJ* ^A152E^ MLC (μg/mL)	Fold Difference (*yfiJ* ^A152E^/*yfiJ* ^+^)
Daptomycin	32.74	32.68	0.99
ECO-02301	0.40	1.46	3.65
LDA*	ND^†^	ND	9.58

*Linearmycins A and B extracted from *S*. Mg1 cultures.

^†^ND, not determined.

Because polyene antibiotics typically exert their effects on the cellular membrane, we wanted to determine if LDA resistant alleles of *yfiJK* provide *B*. *subtilis* with a generalizable cross resistance to membrane-active antibiotics. Daptomycin is a lipopeptide antibiotic that targets the cell membrane [39,40,53]. The killing mechanism of daptomycin is not lytic, although lysis follows prolonged exposure [54]. We found that daptomycin caused a morphologically similar lysis and degradation of *B*. *subtilis* when spotted on a filter paper disc adjacent to a colony (Fig 3). A LDA^R^ strain of *B*. *subtilis* also lysed when exposed to daptomycin. In comparison to the LDA sensitive strain, the LDA^R^ strain showed some residual opacity following lysis, suggesting that LDA^R^ alleles might provide cross-protection to daptomycin (Fig 3). However, we found the MLC of daptomycin was identical between the LDA resistant and sensitive strains (Table 3). Our results suggest that YfiJK signaling provides resistance either specifically to linear polyene molecules related to linearmycins, or commonly to the type of lytic cell damage caused by linearmycins.

### The ABC transporter YfiLMN is necessary for LDA resistance

**Fig 4:**
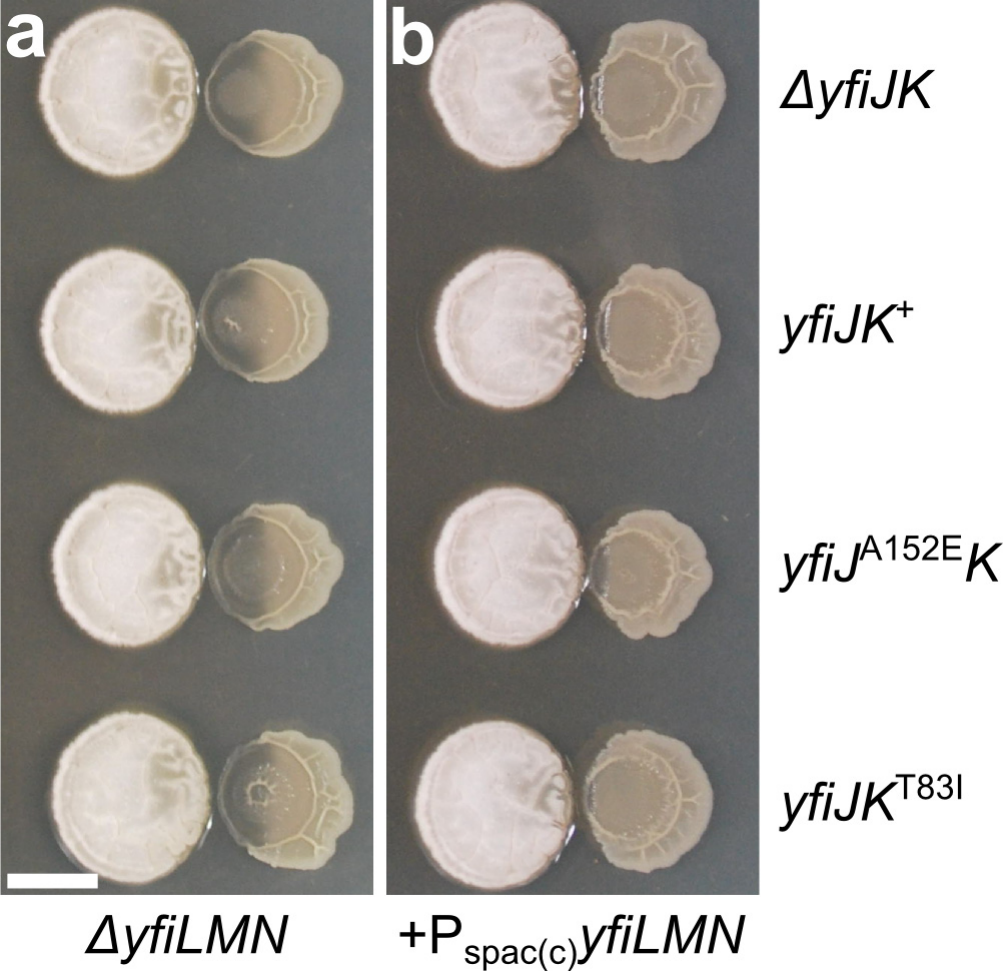
The ABC transporter YfiLMN is necessary for LDA resistance. (a) Strains with deletions of *yfiJKLMN* from *B*. *subtilis* (PDS0653) and *yfiJK* alleles inserted at the *amyE* locus as labeled on the right: *yfiJK* ^+^ (PDS0658), *yfiJ* ^A152E^ *K* (PDS0686), or *yfiJK* ^T83I^ (PDS0660). When cultured with *S*. Mg1, all *B*. *subtilis* Δ*yfiLMN* strains were lysed including those with a LDA^R^ allele of *yfiJK*. (b) Strains with the alleles of *yfiJK* as shown in (a), but containing P_spac(c)_-*yfiLMN* inserted at the *yhdG* locus. All strains were resistant to LDA from *S*. Mg1 with minimal lysis visible next to the *S*. Mg1 colony. LDA resistance was observed in a strain lacking *yfiJK* (PDS0718), a strain with *yfiJK* ^+^ (PDS0719), and in strains with LDA^R^ alleles *yfiJ* ^A152E^ *K* (PDS0720) and *yfiJK* ^T83I^ (PDS0721). All cultures place *S*. Mg1 on the left and *B*. *subtilis* on the right. The cultures were photographed after 72 hours co-incubation on MYM agar plates. All images represent triplicate samples. Scale bar is 5 mm.

Immediately downstream of the *yfiJK* operon are three genes, *yfiLMN*, predicted to encode an ABC transporter [45,55]. This genetic architecture is similar to peptide-antibiotic resistance systems previously characterized in *B*. *subtilis* and other Firmicutes [56]. In these systems, a TCS and an ABC transporter are functionally linked and required for antibiotic resistance. We hypothesized that YfiJK-LMN may function similarly to confer LDA resistance. Thus, we were interested in determining if YfiLMN is necessary for LDA resistance. We engineered a strain with all five genes, *yfiJKLMN*, deleted. The Δ*yfiJKLMN* strain was lysed in co-culture with *S*. Mg1 (Fig 4). We inserted resistant alleles of *yfiJK* at the non-essential *amyE* locus to generate strains unable to produce YfiLMN but possessing LDA^R^ alleles of *yfiJK*. When cultured with *S*. Mg1, these strains were sensitive to LDA (Fig 4). We then complemented the loss of *yfiLMN* in these strains by inserting the *yfiLMN* genes, including the intergenic region between *yfiK* and *yfiL*, at the non-essential *lacA* locus. A predicted terminator exists downstream of *yfiK* (-8.9 kcal/mol) (genolist.pasteur.fr/SubtiList) [57]. Our initial *yfiLMN* complementation construct included sequence immediately downstream of the terminator. However, this construct failed to complement the loss of resistance (S4 Fig). Upon further investigation, we found no recognizable promoter elements in the intergenic region between the *yfiK* terminator and *yfiL* (143 bps). We hypothesized that *yfiJKLMN* may constitute a single operon with some level of terminator read-through resulting in *yfiLMN* expression. To circumvent the lack of an independent promoter, we placed the expression of *yfiLMN* under a constitutive P_spac(c)_ promoter and inserted these constructs at the non-essential *yhdG* locus [58]. Under constitutive expression, the *yfiLMN*-complementation strains were LDA resistant, showing only minimal lysis in co-culture with *S*. Mg1 (Fig 4). This effect was observed even in a strain complemented with wild-type *yfiJK* and in a strain lacking *yfiJK* entirely. These results demonstrate that YfiLMN is necessary for LDA resistance, and that constitutive expression bypasses the need for YfiJK. We speculate that YfiLMN either removes linearmycins from *B*. *subtilis* cells to provide resistance, or alternatively, functions in cell envelope processes or regulatory functions that control LDA resistance. Determining the mechanism of YfiLMN-mediated LDA resistance will require further investigation.

### Intersection of colony developmental phenotypes and LDA resistance in LDA^R^ strains

**Fig 5:**
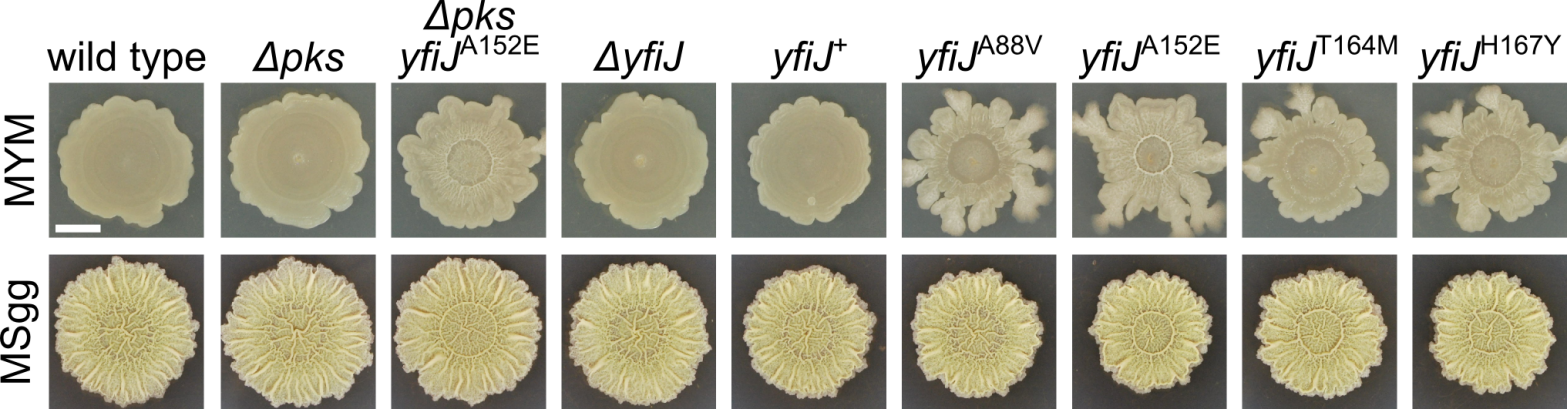
LDA^R^ mutants display aberrant wrinkled morphology. Representative colonies of *B*. *subtilis* with LDA^R^ alleles yfiJ^A88V^ (PDS0575), yfiJ^A152E^ (PDS0572), yfiJ^T164M^ (PDS0573), and yfiJ^H167Y^ (PDS0574) show architecturally complex colonies on MYM, while wild type (PDS0066), Δ*pks* (PDS0067), and Δ*yfiJ* (PDS0555) do not (top panels). The LDA^R^ colonies show both biofilm-like morphology and motile outgrowths at the colony periphery. When cultured on MSgg media, the LDA^R^ mutant strains develop biofilm colony morphology similar to control strains (lower panels). The *Δpks yfiJ* ^A152E^ strain is a representative spontaneous LDA^R^ mutant. All photographs were taken after 72 h. Scale bar is 5 mm.

In our study of the different LDA^R^ alleles, we observed some variation in the degree of wrinkled, motile phenotype in competition with *S*. Mg1 or under LDA exposure. To separate effects of the competitor from inherent LDA^R^ phenotypes, we plated colonies of LDA^R^ strains in isolation to view morphological features. All of the *yfiJK* mutant strains displayed a pattern of increased colony wrinkling and spreading across the agar surface and were distinct from the wild-type strain (Fig 5). We asked if differences in LDA^R^ morphology would be visible on the biofilm-inducing medium, MSgg [59]. The mutant *B*. *subtilis* colonies developed a wrinkled appearance similar to wild type, indicating that traditional biofilm morphology and development are not disrupted in the mutant strains (Fig 5). We also noted that *B*. *subtilis* strains, either wild type or Δ*yfiJ*, formed smooth colonies in the absence of *S*. Mg1 when grown on rich media (Fig 5). In contrast, the same *B*. *subtilis* colonies in competition assays tend so show a somewhat wrinkled morphology, regardless of the *yfiJK* alleles present. Thus, the morphology of the *B*. *subtilis* colonies appears to be influenced by a combination of both the LDA alleles and the presence of the competitor species.

**Fig 6:**
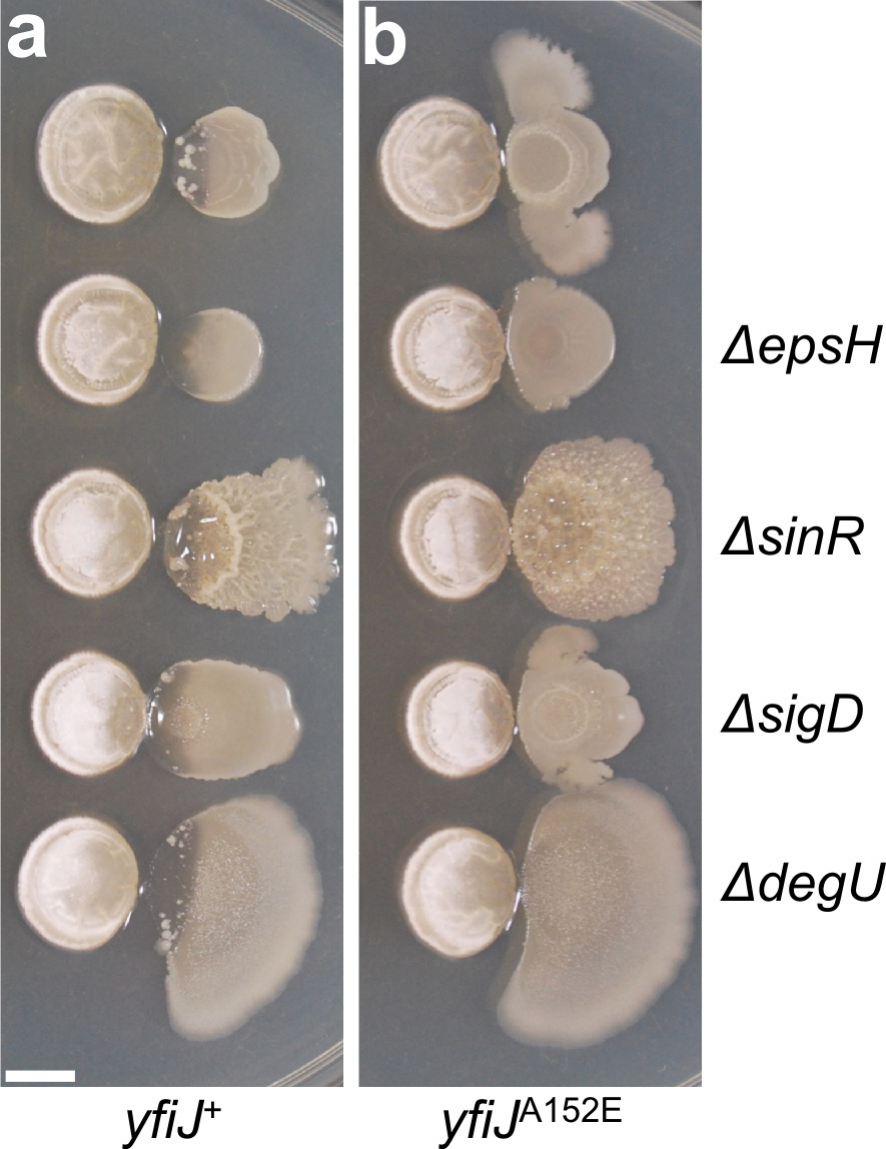
LDA^R^ mutants display a visible response to *S*. Mg1 in addition to inherent colony phenotypes. Wild type and LDA^R^ mutants were spotted in a perpendicular pattern, cross-wise to each other- *B*. *subtilis* (vertical) and *S*. Mg1 (horizontal). (Left) Strains of *B*. *subtilis* with *yfiJ* ^+^ (PDS0571) have flat, immotile colonies. Proximal to *S*. Mg1, the colonies are lysed and degraded. (Right) Strains of *B*. *subtilis* with the LDA^R^ allele *yfiJ* ^A152E^ (PDS0572) develop heterogeneous colonies, having wrinkled surfaces and motile outgrowths. Notably, the colonies of *yfiJ* ^A152E^ *B*. *subtilis* near *S*. Mg1 have a distinctive spreading morphology. Plates were photographed after 96 hours co-incubation on MYM + 1.5% agar. Plates represent duplicate experiments.

To directly compare colony morphology in isolation and with the competitor, the wild type and LDA^R^ strains were cultured at different distances to *S*. Mg1. We inoculated colonies of LDA^R^ *B*. *subtilis* and *S*. Mg1 in a perpendicular, cross-wise pattern on 1.5% agar MYM medium to provide a format for increasing distances between colonies of each species (Fig 6). The growth of *B*. *subtilis* with the wild-type *yfiJ* allele showed smooth colony formation with lysis proximal to the *S*. Mg1. In contrast, the *B*. *subtilis* strain with the *yfiJ* ^A152E^ allele revealed different effects based on its proximity to *S*. Mg1 (Fig 6). The LDA^R^ colonies distant from *S*. Mg1 had the expected wrinkled morphology and spreading outgrowths, as was observed when cultured in isolation (Fig 5). However, the *S*. Mg1-proximal colonies were morphologically different with a flattened surface and more uniform spreading pattern. The observed changes in colony morphology associated with LDA^R^ suggest that the YfiJK TCS regulates both specific resistance and developmental functions that coordinate a survival response to the competitor species.

### LDA^R^ and colony morphology phenotypes are genetically separable

**Fig 7:**
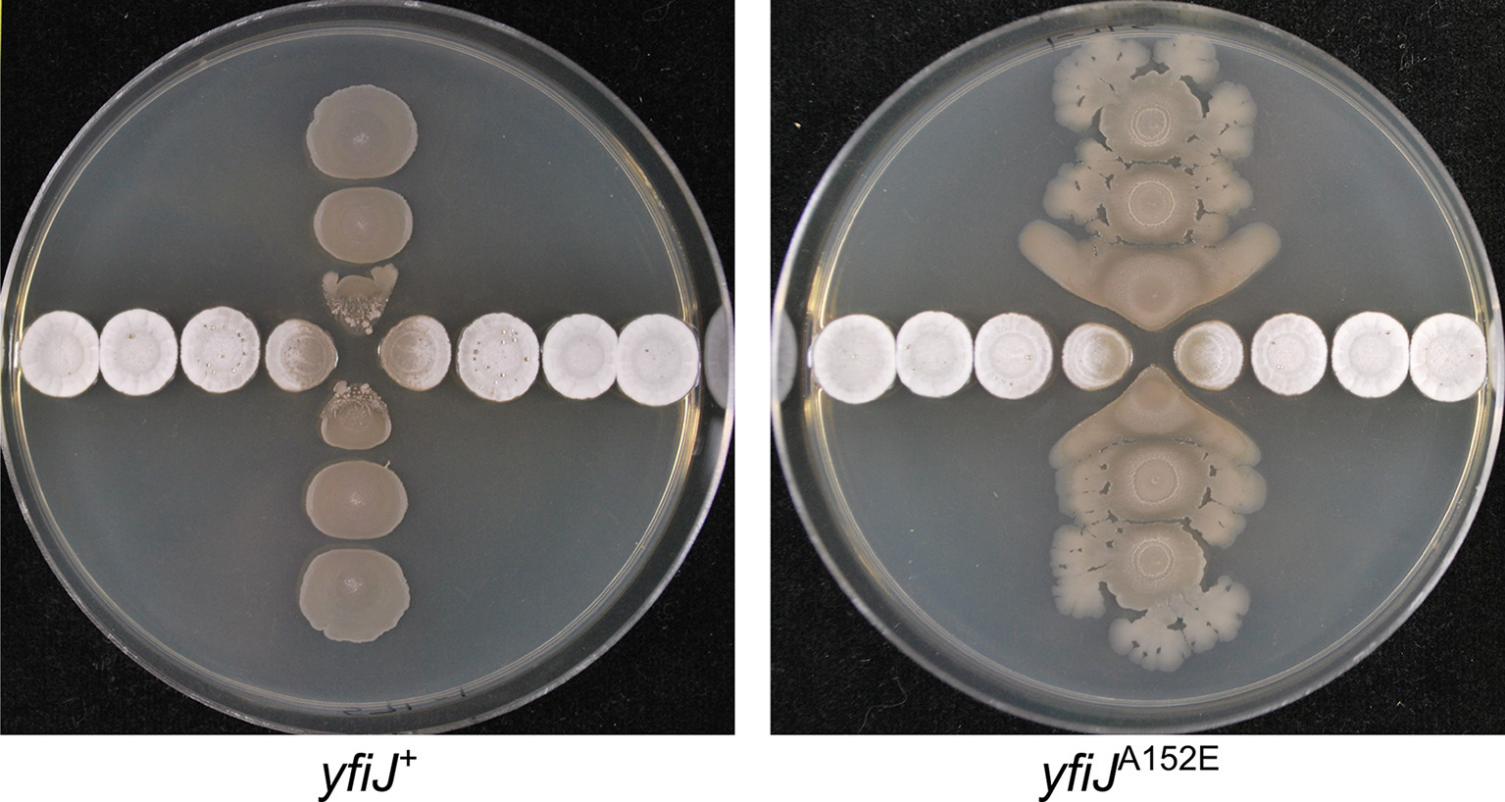
Colony morphology and LDA resistance are separable phenotypes. Genes involved in biofilm formation (*epsH*, *sinR*, and *degU*) and motility (*sigD*) were deleted from strains with either (a) *yfiJ* ^+^ (PDS0571) or (b) the LDA^R^ allele *yfiJ* ^A152E^ (PDS0572). In all cases, the biofilm and motility mutant strains were sensitive to lysis with wild-type *yfiJ* and were resistant to lysis with *yfiJ* ^A152E^. All cultures place *S*. Mg1 on the left and *B*. *subtilis* on the right. Colonies were photographed after 72 hours co-incubation with *S*. Mg1 on MYM medium. Images are representative of triplicate samples. Scale bar is 5 mm.

To gain insight into possible connections between colony phenotypes and resistance to lysis, we considered that changes to extracellular matrix (ECM), the associated biofilm-like colony morphology, and changes in motility, may be responsible for LDA resistance [10,60]. For instance, the ECM may impede access of linearmycins to their target, possibly through overproduction of EPS or other matrix components [60]. To test whether LDA resistance is dependent on known components of biofilm ECM, we sought to separate the two processes. We generated an ECM-defective strain, which was unable to produce exopolysaccharide (EPS) due to an *epsH* deletion [59], in an otherwise LDA resistant background (*yfiJ* ^A152E^). This strain developed as a flat, mucoid colony, but remained LDA resistant in co-culture with *S*. Mg1 (Fig 7). Based on this result, we concluded that, while EPS production is necessary for the wrinkled colony morphology, intact biofilm matrix in the LDA^R^ strains is not responsible for the LDA resistance mechanism. However, LDA resistance may require other biofilm matrix components [61]. We asked whether hyperactivation of biofilm production would mimic the LDA resistance phenotype. We deleted the gene encoding *sinR*, the master biofilm repressor [62], in a LDA sensitive strain. When *sinR* is deleted, *B*. *subtilis* overproduces biofilm matrix and the colonies grow with a profoundly wrinkled appearance [62]. If biofilm formation is responsible for LDA resistance, then a *ΔsinR* mutant should be resistant in co-culture with *S*. Mg1. However, the *ΔsinR* strain was sensitive to lysis (Fig 7). LDA resistance was observed in a Δ*sinR* strain only in the presence of the mutant *yfiJ* (*yfiJ* ^A152E^).

Biofilm formation is controlled not only by SinR but also by the TCS DegSU. This TCS is responsible for control of the production of biofilm extracellular matrix components. Among these components are BslA and γ-poly-DL-glutamate (γ-PGA) [63–65]. To test if matrix functions provided by DegU may contribute to LDA resistance, we deleted *degU* in a LDA resistant background (*yfiJ* ^A152E^) and cultured the strain with *S*. Mg1. This mutant developed as a flat colony that was LDA resistant, suggesting that resistance to LDA does not require functions provided by DegU (Fig 6). Based on this finding and our SinR and EpsH experiments, we conclude that the changes in colony morphology of LDA^R^ mutants are not the principle cause of LDA resistance.

In addition to wrinkled colony morphology, the enhanced motility of LDA^R^ strains may be linked to resistance. For instance, swarming motility has been associated with elevated antibiotic resistance in multiple bacteria [10]. Previously, we observed lysis in a Δ*sigD* strain, which is deficient in swarming and autolysin production [51,66,67] (S3 Fig). We tested whether a Δ*sigD*, *yfiJ* ^A152E^ double mutant strain would undergo lysis despite the presence of the LDA^R^ allele (Fig 7). This strain maintained both LDA resistance and morphological changes, including colony spreading. The spreading phenotype in the absence of *sigD* is consistent with LDA^R^ mutants exhibiting sliding motility when cultured with *S*. Mg1 [68]. In sum, the combined phenotypes of LDA^R^ support a model wherein activation of YfiJK leads to LDA resistance through YfiLMN activation coordinated with separable changes in colony motility and morphology that promote survival during competition.

### Genes identified by differential expression in a LDA^R^ strain

In an effort to identify a regulatory network for YfiJK, we sought to identify genes differentially expressed in a LDA^R^ mutant that may contribute to colony phenotypes. To perform differential expression analysis, we isolated and sequenced RNA from *yfiJK* ^+^ (PDS0627) and *yfiJ* ^A152E^ *K* (PDS0685) strains cultured on agar plates. In our analysis, we identified six genes with statistically significant changes in expression between the two strains (Table 4). Expression of *yfiLMN* was increased on average ~18-fold in the LDA^R^ mutant. To corroborate this result we used qRT-PCR and observed a ~20-fold increase in *yfiL* expression from the *yfiJ* ^A152E^ *K* strain (S6 Fig). We did not observe a change in expression of *yfiJK* in our RNA-seq experiments, which is consistent with an additional control element between *yfiK* and *yfiL*. Three other differentially expressed genes were all decreased in the LDA^R^ mutant: *des*, which encodes a phospholipid desaturase responsible for cold shock adaptation [69,70] and *yvfRS*, which encodes an ABC transporter of unknown function [55]. Surprisingly, no genes in the *eps* operon or other known biofilm-related genes were identified as differentially expressed between the LDA sensitive and LDA^R^ strains. Also of note, we found no correspondence between the YfiJK-regulated genes we identified by RNA-seq and the regulon previously defined by microarray study of *yfiK* overexpression [47]. In the absence of a clear connection to established biofilm and motility functions, the RNA-seq results suggest that the morphological changes observed in LDA^R^ colonies may arise directly from activation of YfiLMN function combined with repression of *des* (phospholipid content) and *yvfRS* (unknown function) by an unknown mechanism. Alternatively, the morphological changes may occur only in a subpopulation of cells insufficient to be detected during our analysis.

**Table 4:** Differential expression analysis between *yfiJ* ^A152E^ *K* and *yfiJK* ^+^.

Gene	Fold Difference (*yfiJ* ^A152E^ *K*/*yfiJK* ^+^)	FDR
*yfiN*	21.28	2.02^−22^
*yfiM*	19.15	1.70^−20^
*yfiL*	14.14	3.59^−17^
*des*	0.20	3.05^−7^
*yvfR*	0.15	2.54^−7^
*yvfS*	0.14	1.81^−5^

Differential gene expression between a *yfiJ* ^152E^ *K* strain (PDS0685) and a *yfiJK* ^*+*^ strain (PDS0627). Fold differences > 1 indicate increased expression in the *yfiJ* ^A152E^ *K* strain relative to the *yfiJK* ^+^ strain.

### YfiJK is required for transient LDA resistance and small colony formation

**Fig 8:**
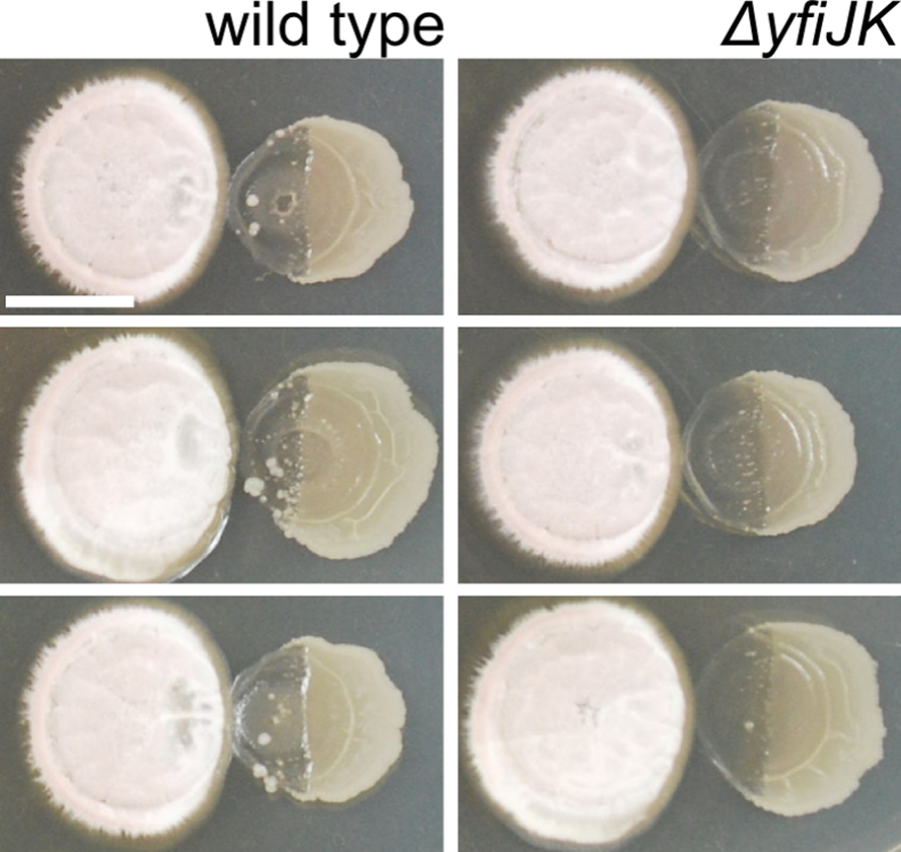
Small colonies among lysed cells in wild-type but not *ΔyfiJK* colonies. Eighteen wild-type and *ΔyfiJK* colonies of *B*. *subtilis* were cultured with *S*. Mg1. Many small, potentially LDA^R^, colonies appeared in the region of lysis of wild-type colonies, while few could be seen in the Δ*yfiJK* strain. The few small colonies observed in the zone of lysis for Δ*yfiJK* did not have the morphological features of the wild-type colonies. Note, S5 Fig shows all eighteen replicate colonies for each strain. All cultures place *S*. Mg1 on the left and *B*. *subtilis* on the right. Photographs were taken after 96 hours co-incubation on MYM agar. Scale bar is 5 mm.

One of our initial goals was to identify mechanisms of resistance in an attempt to expose mechanistic aspects of linearmycin activity. We considered that LDA resistance may only exist under aberrant conditions, which arise through mutations that hyperactivate the YfiJK signaling system. In the absence of a clear phenotype for deletion of the genes, we sought an approach to identify wild-type YfiJK function in colony morphology and LDA resistance. We returned to an early observation that small colonies resistant to LDA emerge in the lysed region of a *B*. *subtilis* colony. The majority of the isolated LDA resistant colonies isolated were only transiently resistant (50/60). We reasoned that if the natural function of YfiJK is to provide temporary resistance to LDA-induced damage, the emergence of transient resistance would depend upon the function of YfiJK. Therefore, we tested 6 independent colonies, each in triplicate, of wild type and Δ*yfiJK* versus *S*. Mg1 to determine if resistant colonies would emerge in the absence of YfiJK (Figs 8 and S5). The resulting cultures showed many small colonies arising in the lysed areas of the wild-type *B*. *subtilis* colonies. By contrast, the few small colonies observed with the *ΔyfiJK* strain did not grow appreciably and lacked the morphological features of the *yfiJK* ^*+*^ colonies (Fig 8). This result is consistent with the natural function of YfiJK providing transient resistance to LDA-induced stress. In the case of *yfiJK* gain-of-function alleles, the substitutions in YfiJK may lock the TCS into an active state wherein every cell becomes resistant to LDA in contrast to the subpopulations observed among wild-type cells. Intriguingly, the transient resistance appears in only a subset of cells in the colony. Variable antibiotic resistance among a clonal population of cells has been described as heteroresistance, and is thought to be advantageous for survival of bacteria during antibiotic treatment [71–73]. The ability to activate YfiJK in a subset of cells may constitute a mechanism of transient heteroresistance to linearmycins and related molecules, but defining the mechanism and limitation to a subpopulation of cells will require further investigation. The observed pattern of YfiJK-dependent LDA resistance highlights that this TCS, and possibly many TCS, may transiently serve a subset of cells in a population during times of competitive crisis.

## Discussion

**Fig 9:**
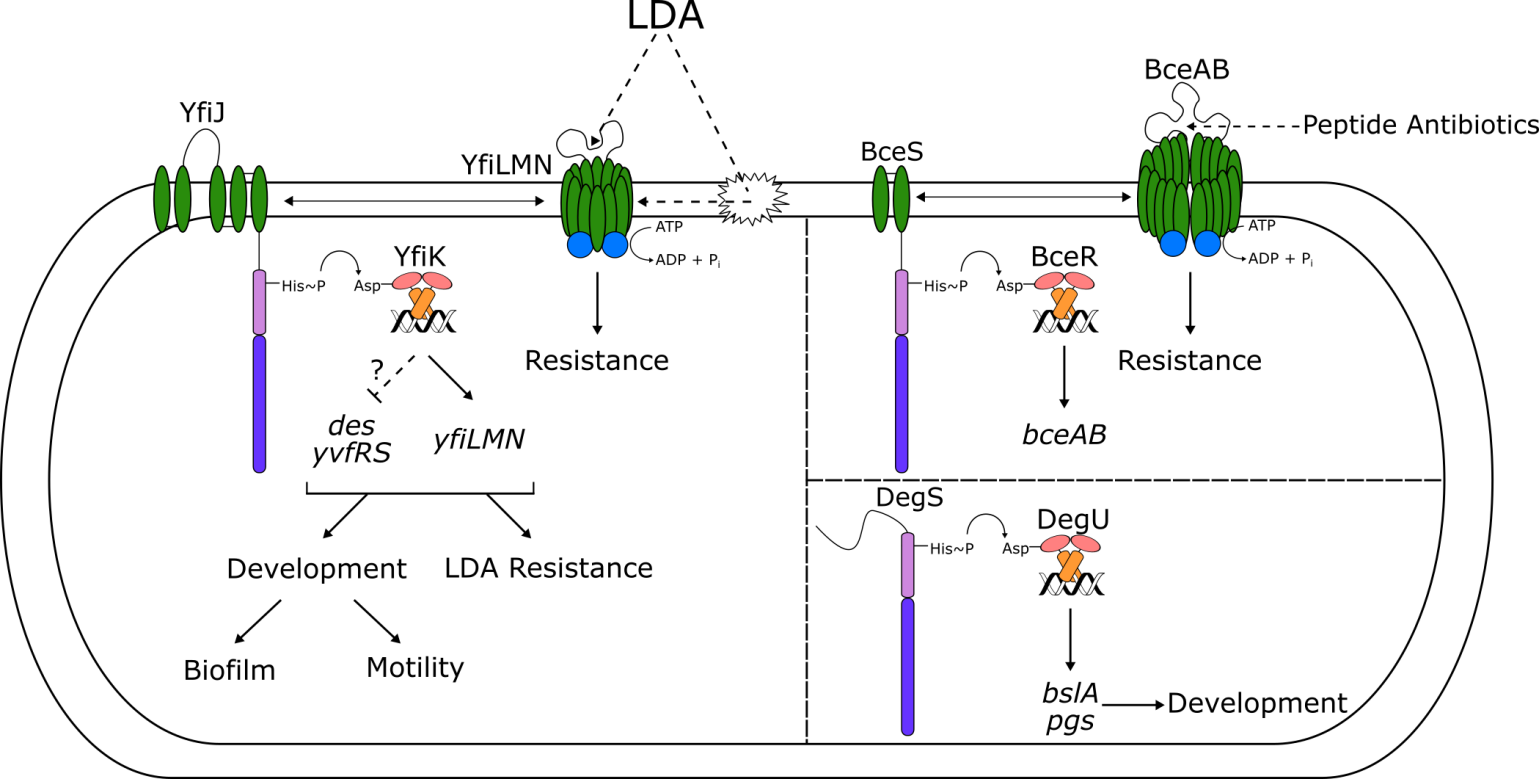
Model for YfiJK-LMN functions in LDA resistance and development. LDA is sensed either directly by the ABC transporter YfiLMN, similarly to the ABC transporter BceAB and peptide antibiotics, or indirectly as membrane damage. This signal is transferred to the histidine kinase YfiJ, which then activates YfiK via phosphorylation. YfiK~P then activates the transcription of *yfiLMN* and likely represses *des* and *yvfRS*, leading to LDA resistance, biofilm formation, and motility through an unknown mechanism. These functions promote survival of *B*. *subtilis* under competitive stress. The YfiJK system differs structurally and functionally from other TCS that control either antibiotic resistance, such as the BceRS-AB system, or development, such as the DegSU system. Interactions between HKs and ABC transporters are shown with double-headed arrows. Hypothesized interactions of molecules with ABC transporters or membranes are shown with dashed arrows.

In this study, we used a two-species culture model of bacterial competition to identify functions that contribute to bacterial competitive fitness. The present study stemmed from an earlier observation of lysis and degradation of *B*. *subtilis* colonies when cultured adjacent to *S*. Mg1 [29]. Here, we first identified linearmycins, produced by *S*. Mg1, as the primary cause of progressive lysis and colony degradation. The culture format used for competition revealed small *B*. *subtilis* colonies spontaneously resistant to lysis. When isolated, the resistant colonies showed a biofilm-like appearance with increased wrinkled colony morphology and aberrant motility. We sequenced whole genomes of the resistant colonies and identified mutations that confer resistance. Genomic analysis revealed alleles of the *yfiJK* operon, which encodes a two-component system of previously unknown function. Based on our observations, we define *yfiJK* as a regulator of *yfiLMN*, encoding an ABC transporter, and possibly other target genes that govern modes of colony growth and motility (Fig 9).

We show that the LDA resistance is not dependent upon known biofilm-specific functions, suggesting that colony morphology and LDA^R^ are separable processes, unified under YfiJK regulation. Two-component systems are well established as regulators for cellular responses to environmental stresses, including antibiotics [74,75]. The significance of the current work is the use of model interspecies competition to reveal both the agent of aggression, linearmycins, and a multifaceted survival response from genes with no prior functional assignment, *yfiJKLMN*.

Only gain-of-function mutations in *yfiJK* were identified in this study to cause LDA resistance. The resistance alleles of *yfiJK* were due to missense mutations causing changes to four regions of YfiJK: *(i)* the third TM helix in YfiJ, *(ii)* the cytoplasmic linker between the fifth TM helix and the dimerization and histidine phosphotransfer (DHp) domain in YfiJ, *(iii)* the C-terminal end of the DHp, and *(iv)* the regulator domain in YfiK. We hypothesize that each of these amino acid substitutions are responsible for conformational changes in YfiJK, leading to a constitutively active state. A previous study described a similar phenotype caused by point mutations of *pmrAB* in *Pseudomonas aeruginosa*. Gain-of-function alleles in *pmrB* lead to polymyxin B resistance via increased signaling through the histidine kinase [76]. We also considered an alternative mechanism, wherein point mutations in *yfiJK* could lead to non-cognate interactions of YfiJ or YfiK and aberrant signal transduction [77]. However, we view this mechanism as unlikely because only one of the affected residues (L254) lies in the DHp domain, which is predicted to be involved in specificity [78], and LDA resistance required the presence of the phosphoacceptor residue in the cognate partner. Thus, we conclude that gain-of-function alleles cause LDA resistance and changes in both colony morphology and motility, and that the signaling is specific to YfiJK. Although the specific defects caused by each allele will require further investigation, we note that many of the mutations we observed are responsible for amino acid changes in the cytoplasmic linker of YfiJ. The cytoplasmic linker domain of HKs has been best characterized in periplasmic-sensing histidine kinases. In these kinases, the linker may contain conserved PAS or HAMP domains that are necessary for signal transduction from the sensory machinery to the kinase domains [46,79–81]. YfiJ has neither of these conserved domains, suggesting that the short linker in this protein is the sole signal-transducing domain. The mutations in the *yfiJ* linker, through fixing the protein in activated state, may be very informative for determining the mechanism of signal transduction via the YfiJ intramembrane histidine kinase.

Two-component systems are commonly involved in sensing antibiotic and environmental stress [74,75]. Among Firmicutes, a conserved mechanism for resistance to peptide antibiotics pairs genes for two-component systems and ABC transporters [56,82,83]. The identification of mutations in *yfiJK* suggests the cell envelope is the linearmycin target, based on comparison to other TCS-ABC transporter pairs in *B*. *subtilis* [56]. Immediately downstream of *yfiJK* are three genes, *yfiLMN*, that are predicted to encode an ABC transporter. We found that when *B*. *subtilis* was unable to produce YfiLMN, the colonies were LDA sensitive and failed to develop altered colony morphology, regardless of the presence of a LDA^R^ allele of *yfiJK*. Furthermore, expression of *yfiLMN* under a constitutive promoter resulted in LDA resistance, even in the absence of *yfiJK*. Thus, the YfiLMN transporter is necessary and sufficient for LDA resistance. We hypothesize that YfiLMN may act as an exporter either for linearmycin or for cell envelope remodeling factors that lead to LDA resistance.

We used RNA-seq to identify genes that may be regulated by YfiJK. As expected we identified that *yfiLMN* expression was increased in a LDA^R^ mutant. We also identified *yvfRS*, encoding an ABC transporter of unknown function, and *des* as genes downregulated by YfiJK. The *des* gene encodes a fatty acid desaturase that is responsible for altering membrane fluidity in response to cold shock [69,70]. Intriguingly, *B*. *subtilis* strains with *des* deletions are more susceptible to daptomycin-treatment, potentially due to their altered membrane fluidity [84]. Antifungal polyenes structurally related to linearmycins target ergosterol in fungal membranes [39–42]. The decreased expression of *des* in LDA^R^ mutants may contribute to resistance by affecting interactions between linearmycins and the cell membrane. Characterization of the cell envelopes of LDA sensitive and LDA^R^ strains may provide insight into the mechanism of linearmycin-induced lysis.

Mutants with LDA^R^ alleles of *yfiJK* grow as rugose colonies that resemble some aspects of biofilm development on rich media, which does not support traditional biofilm development. We demonstrated that we could functionally divorce this colony morphology phenotype and LDA resistance by expressing *yfiLMN* constitutively and by introducing deletions of genes specifically required for biofilm development (*epsH*, *sinR*, *degU*). In so doing, we found that changes to the biofilm extracellular matrix are not responsible for resistance. LDA resistance may be modulated by specific matrix or cell envelope modifications activated by YfiJK-LMN, but such modifications remain to be identified. Although we found no obvious candidates in our RNA-seq data to explain colony morphological changes, the decreased expression of *des* or *yvfRS* may contribute to alterations in colony development. We also observed that LDA^R^ mutants respond to *S*. Mg1 by inducing motility, whereas wild type *B*. *subtilis* colonies are lysed. The pleiotropic phenotypes of *yfiJK* LDA^R^ alleles differentiate this coupled TCS-ABC transporter system from the BceRS-AB, PsdRS-AB, YxdJK-LM systems in *B*. *subtilis*, which appear to be dedicated antibiotic resistance systems [56,85–88]. To our knowledge, there are no phenotypes associated with development that have been attributed to these TCS-ABC transporter pairs, suggesting that YfiJK holds a specialized role in providing specific LDA resistance and in activating biofilm development and motility, both of which are known to increase resistance to antimicrobials [10,60]. We propose that activation of YfiJK-LMN promotes competitive fitness of *B*. *subtilis* by coupling a specific resistance mechanism (LDA^R^) with generalized-resistance that occurs as a consequence of altered development and motility. A recent study using strains of *Pseudomonas aeruginosa* demonstrates that biofilm formation is stimulated in response to competition, as opposed to a cooperative function of different strains or cell types [9]. The identification of YfiJK as a regulator of biofilm and motility functions is consistent with a model wherein competition with *S*. Mg1 induces developmental responses, including biofilm and colony spreading, among a subpopulation of resistant cells of *B*. *subtilis*.

Using microbial competition we assigned resistance and developmental functions to a previously uncharacterized TCS in *B*. *subtilis*. Without imposing the conditions of competition on *B*. *subtilis*, these TCS functions may be difficult to identify, because the *yfiJ*, *yfiK*, and *yfiJK* deletion mutants have no phenotype when compared to wild type. The *B*. *subtilis* genome encodes 36 histidine kinases and 34 response regulators [89]. The functions of at least 11 of these TCS are currently unknown. Bacteria use these systems to sense and respond to their environment, which include stresses and nutrient conditions, but also include other bacteria and their antagonistic enzymes and specialized metabolites. Many TCS of unknown function may have a role in the context of microbial competition, despite having no distinct phenotype under laboratory conditions. Thus, microbial competition studies provide an effective approach to identify functions for TCS and other genes that promote competitive fitness of bacteria. By expanding our knowledge of individual competitive functions, a more comprehensive view of bacterial competitive fitness will emerge.

## Materials and Methods

### Bacterial strains, media, and general cloning

The strains of *B*. *subtilis* we used in this study are listed in S3 Table. We cultured *B*. *subtilis* strains at 37°C in lysogeny broth (LB) [1% tryptone (Bacto), 0.5% yeast extract (BBL), 0.5% sodium chloride (Sigma)] or on LB agar plates [1.5% Agar (Bacto)]. We maintained *Streptomyces* sp. Mg1 (PSK0558) as a spore stock in water at 4°C. Unless otherwise stated all co-cultures were grown on MYM [0.4% malt extract (Bacto), 0.4% yeast extract (BBL), 0.4% D-(+)-maltose monohydrate (Sigma)] with 2% agar (Bacto). We used chloramphenicol (5 μg/mL), kanamycin (5 μg/mL), MLS (1 μg/mL erythromycin, 25 μg/mL lincomycin), spectinomycin (100 μg/mL), and tetracycline (20 μg/mL) as needed. The primers we used in this study are listed in S4 Table. We used *Escherichia coli* DH5α or XL-1 blue for plasmid maintenance and manipulation. We prepared All *B*. *subtilis* genetic manipulations in either the 168 or PY79 strain background and then transduced them to NCIB3610 using SPP1 phage transduction as previously described [90].

### LDA extraction and identification

We wetted 1 g Diaion HP-20 resin in 25 mL methanol (MeOH) followed by washes with 25 mL of water five times while shaking. Next, we removed the bulk liquid and resuspended the resin in 250 mL of MYM. We sterilized the media and resin by autoclaving the mixture. We inoculated cultures using 1 mL of *S*. Mg1 that was grown overnight (10^7^ spores in 3% tryptone soy broth). We cultured the *S*. Mg1 for 6 d at 30°C while shaking at 225 RPM in the dark. We performed all culture growth and extractions in low ambient light, because the activity of extracts was diminished or lost if manipulated in the light. We separated the HP-20 resin from the bulk of the *S*. Mg1 by repeatedly washing the resin with water until all visible filaments were removed. To extract resin-bound molecules, we washed the resin with successive 25 mL volumes of MeOH until the solvent was clear. To generate our crude extract, we pooled the washes and removed MeOH using a rotary evaporator. The crude extract was dissolved to 100 mg/mL in 50% acetonitrile (ACN) and fractionated over a C_8_ solid-phase extraction (SPE) column eluted with a MeOH/water stepwise gradient. We removed solvent from our fractions using a rotary evaporator and suspended the fractions to 50 mg/mL in 50% ACN. We tested the fractions for lytic activity against *B*. *subtilis* by spotting 10 μL on a filter paper disc adjacent to a *B*. *subtilis* colony that had been pre-grown for 24 h and observing lysis over a period of 48 h. The 70% and 80% MeOH fractions were active in the lysis assay and pooled for further fractionation.

Using an Agilent 1200 HPLC system, we further fractionated the active extract fractions over a semi-preparative (10 x 250 mm, 5 μm) Phenomenex Luna C_18_ column and eluted with an ACN/20 mM ammonium acetate pH 5 (NH_4_OAc) gradient running at 5 mL/min. The elution program was as follows: 1) 5 min at 40% ACN then 2) a gradient up to 50% ACN over 10 min then 3) a gradient up to 75% ACN over 5 min, and 4) a gradient diminishing to 40% over 5 min. We injected 35 μL of pooled active fraction per injection. We collected time based fractions and tested them for lytic activity as above. Active fractions were analyzed by mass spectrometry using a Bruker microTOF mass spectrometer. For NMR analysis, the sample was dried and resuspended in 300 μL deuterated dimethylsulfoxide (DMSO-d_6_). We collected spectra on a Bruker Avance III 500 MHz spectrometer equipped with a cryoprobe.

### LDA resistant mutant isolation and whole genome sequencing

We diluted overnight cultures inoculated with a single colony of *B*. *subtilis Δpks* (PDS0067) into 5 mL of LB at OD_600_ = 0.08 with no antibiotics. We cultured the cells to early stationary phase (OD_600_ = 0.9–1.5) at 37°C and spotted 2 μL on MYM7 plates [as above with 100 mM MOPS and 25 mM potassium phosphate buffer pH 7, 1.5% agar (Bacto)]. After 24 h incubation, we placed 6 mm filter paper discs next to the *B*. *subtilis* colonies and added 10 μL of extract from *S*. Mg1. We returned the plates to the incubator and observed lysis and colony degradation over the next 48 h. After incubation, small colonies were observed in the region of lysis. We isolated 60 small colonies and passaged them on LB plates. We tested each isolate for LDA resistance using co-culture, as described below.

LDA^R^ mutants that were stable through passage in isolation and the parental *Δpks* strain were used for whole genome sequencing. Sequencing libraries were prepared using the PCR-free TrueSeq Kit from Illumina. 250 bp paired-end reads were sequenced using an Illumina MiSeq. We mapped reads from the LDA^R^ mutants onto the parental *Δpks* strain using MIRA and identified mutations by consensus discrepancy between the sequences [91,92].

### Construction of *yfiJK* and *yfiJKLMN* deletion mutants

We used long-flanking homology PCR to delete *yfiJK* and *yfiJKLMN*. Briefly, to delete *yfiJK* we amplified the upstream sequence using primers 13 and 14, the downstream sequence using primers 15 and 16, and the kanamycin resistance cassette from pDG780 using primers kn-fwd and kn-rev. We mixed the three PCR products together and used primers 13 and 16 to amplify a product, which we directly transformed into PDS0312 to generate PDS0546.

To delete *yfiJKLMN* we used primers 76 and 77 to amplify the upstream sequence of *yfiJ* and primers 78 and 79 to amplify the downstream sequence of *yfiN*. We combined these fragments with the kanamycin resistance cassette and amplified a product using primers 76 and 79, which we directly transformed into PDS0312 to generate PDS0652.

### Complementation of *yfiJ* and *yfiJK*

To test alleles of *yfiJ*, we complemented the *ΔyfiJ* deletion. We amplified *yfiJ* with primers 25 and 26 from wild type and spontaneous LDA^R^ mutants. These primers include a BamHI and EcoRI site, which we used to clone the product into plasmid pDR183 (*lacA*::*mls*). We transformed the plasmids into PDS0559 and verified insertion into the *lacA* locus by PCR. We moved these constructs into PDS0555 using SPP1 phage transduction.

We tested alleles of *yfiK* by complementing both *yfiJK* together into a *ΔyfiJK* strain. We complemented both genes together because *yfiK* is the second gene in the operon. We amplified *yfiJK* using primers 54 and 75 from wild-type or spontaneous LDA^R^ mutant and the plasmid backbone of pDR111 (*amyE*::*spc*, without the IPTG-inducible system) using primers 59 and 74. We combined these products together using Gibson assembly [93], transformed the plasmid into PDS0546, and verified insertion into the *amyE* locus by PCR. We moved these constructs into PDS0554 using SPP1 phage transduction.

### Complementation with P_spac(c)_ *yfiLMN*

To complement *yfiLMN* we first amplified P_spac(c)_ from BJH157 using primers 112 and 113. These primers included an EcoRI and SpeI site, which we used to clone the P_spac(c)_ fragment into pBB275 to generate pRMS1. We amplified *yfiLMN* using primers 120 and 121 and the backbone of pRMS1 using primers 118 and 119. We assembled these fragments using Gibson assembly and transformed them directly into PDS0652 to generate PDS0717.

### Site-directed mutagenesis

We used primer-mediated site-directed mutagenesis to generate phosphoacceptor residue changes. To generate *yfiJ* ^H201N^ alleles we used primers 42 and 43. To generate *yfiK* ^D54A^ alleles we used primers 50 and 51. Briefly, we PCR amplified plasmids containing *yfiJ* or *yfiJK* using overlapping primer pairs that included a single nucleotide change, DpnI-digested the reactions, and transformed *E*. *coli*. We isolated the plasmids and sequenced them to verify the mutation. We used plasmids containing the mutations to transform *B*. *subtilis* as above.

### Lysis co-culture assays

To observe lysis, we grew cultures of *B*. *subtilis* as above and spotted 1 μL of *B*. *subtilis* on 20 mL MYM plates. We then spotted 5 μL of a 10^9^ spores/mL stock of *S*. Mg1 ~6 mm from *B*. *subtilis*. These plates were incubated at 30°C and monitored every 24 h.

### Motility co-culture assays

To observe the effect of *yfiJ* alleles on motility we used a modified version of a motility assay we previously described [18]. We plated 2.5 μL of a 10^7^ spores/mL stock of *S*. Mg1 on a 25 mL MYM plate and incubated the plate at 30°C. After 12 h of growth, we spotted 1.5 μL of *B*. *subtilis*, grown as above, perpendicularly to *S*. Mg1, returned the plates to the 30°C incubator, and monitored the plates every 24 h.

### Measuring minimum lytic concentrations

To measure MLC values we used an agar diffusion assay. We grew cultures of a LDA sensitive strain (PDS0571) and a LDA^R^ strain (PDS0572) in 25 mL of MYM to OD_600_ = 2. When the cultures reached this density, we centrifuged the cultures at 3220 x g for 5 min and resuspended the cell pellet in half the volume to reach OD_600_ = 4. We mixed 1.5 mL of resuspended cells with 4.5 mL of MYM agar (0.67%) and poured the layer over a MYM plate to generate an overlay with OD_600_ = 1 and 0.5% agar. We placed 6 mm filter paper discs onto the overlay and added 10 μL of 2-fold serial dilutions of daptomycin, ECO-02301, and LDA to the discs. Afterwards we incubated the plates for 4 h at 30°C and then photographed the plates. We measured haloes of lysis using ImageJ [94] and determined MLC values by plotting natural log-transformed antibiotic concentrations versus the area of lysis, and calculated the intercept to determine MLC values for the lytic agents [95].

### RNA-seq

We grew two independent cultures each of *yfiJK* ^+^ (PDS0627) and *yfiJ* ^A152E^ *K* (PDS0685) strains as above. When the cultures reached early stationary phase we diluted them 10^−3^ in LB and spread plated 100 μL on MYM plates. After 24 h we scraped the lawns of *B*. *subtilis* into RNAprotect Bacteria Reagent (Qiagen) and isolated RNA using an RNeasy mini kit (Qiagen). We removed trace DNA from the RNA samples using a Turbo DNA-free kit (Applied Biosystems). The ribosomal RNA was removed from RNA samples using a Ribo-Zero rRNA Removal Kit (Gram-Positive Bacteria) (Illumina). 50-bp single-end reads libraries were prepared using a TruSeq Stranded Total RNA Kit (Illumina) and sequenced on an Illumina HiSeq 2500. We mapped reads to each open reading frame (ORF) in the *B*. *subtilis* 168 genome (GenBank: NC_000964.3) with kallisto [96] and used edgeR [97] for differential gene expression analysis. We filtered out lowly expressed ORFs (<1 count per million and only represented in one of the four samples) and used trimmed mean of M-value normalization to calculate effective library sizes before analysis [98]. We used the single-factor exact test and reported differentially expressed genes with a false discovery rate cutoff of < 1^−4^ [99]. The raw reads for this experiment are accessible from NCBI BioProject Accession PRJNA29593.

### Quantitative RT-PCR (qRT-PCR)

We isolated RNA from PDS0627 and PDS0685 as above and preformed qRT-PCR similarly as previously described [18]. Briefly, we used 100 ng of total RNA as template for cDNA synthesis using a High-Capacity RNA-to-cDNA Kit (ThemoFisher Scientific). We used an SsoAdvanced Universal SYBR Green Supermix Kit (Bio-Rad) for and preformed quantitative PCR with a CFX96 Touch real-time PCR thermocycler (Bio-Rad). We used the following cycling parameters: denaturation at 95°C for 30 s; 40 cycles of denaturation at 95°C for 15 sec, annealing at 58°C for 30 s, and extension at 72°C for 30 s; and a final melting curve from 60°C to 95°C for 6 min. We used *gyrB* as our reference gene. We amplified *yfiL* using primers q1 and q2 and *gyrB* using primers *gyrB* qPCR-fwd and *gyrB* qPCR-rev (S3 Table). We ran each reaction in triplicate. Using LinReg [100] we calculated primer efficiency and quantification cycle values. We normalized *yfiL* abundance to *gyrB* and report fold difference relative to PDS0627.

## Supporting Information

S1 Fig Characterization of the molecule responsible for LDA. (a) Mass spectrum of the isolated peak with lytic activity against *B*. *subtilis*. The prominent masses detected in the experiment match those of linearmycin B, *m/z* 594.9 [M+H+Na]^2+^, 605.8 [M+2Na]^2+^ and 1167.7 [M+H]^+^ (b) Structure of linearmycin B with ^13^C NMR chemical shift assignments obtained in DMSO-d_6_. Carbons are numbered linearly starting with the carbonyl carbon of the carboxylic acid group.
(TIFF)

S2 Fig Disruption of the linearmycin biosynthetic gene cluster abolishes LDA. The *S*. Mg1 wild type strain (a) and a strain with a chromosome arm deletion (*Δ37*) that includes the linearmycin biosynthetic gene cluster (b) were co-cultured with *B*. *subtilis Δpks* (PDS0067) (top left panels). *Bacillus subtilis* is not lysed by *S*. Mg1 *Δ37*. Extracts from each streptomycete were spotted on filter paper discs adjacent to a *B*. *subtilis Δpks* colony (lower left panels). The *B*. *subtilis* colony challenged with the *S*. Mg1 *Δ37* extract was not lysed. The extracts were analyzed by HPLC (right panels). Linearmycins are detected by UV absorbance at 333 nm (blue) while the background is shown by the 254 nm absorbing trace (red). The predominant difference in the extracts is the presence or absence of linearmycins A and B. Linearmycin A (*m/z* 1140) and B (*m/z* 1166) identities were confirmed by mass spectrometry. Colonies were photographed after 72 h of co-incubation or after 48 h exposure to extract. Scale bar is 5 mm.
(TIFF)

S3 Fig Genes predicted to be repressed by YfiK are not responsible for LDA. Spore-killing factor (SKF) and autolysis were predicted to be regulated by YfiK. Strains of *B*. *subtilis* (right) with deletions in genes responsible for SKF biosynthesis (*ΔskfA-H*) (DL598), an autolysin inhibitor (*ΔiseA*) (PDS0785), deletions in the major autolysin regulator σ^D^ (*ΔsigD*) (DS323), and deletions in three major autolysins (*ΔlytABC*, *ΔlytD*, *ΔlytF*) (DS2483) were tested for resistance to LDA in co-culture with *S*. Mg1 (left). All strains lysed similarly to wild type (PDS0066). Cultures were photographed after 72 h co-incubation on MYM agar plates. Scale bar is 5 mm. These results were consistent across six replicates.
(TIFF)

S4 Fig Complementation of *yfiLMN* with *yfiK* terminator-*yfiL* intergenic sequence fails to restore LDA resistance. The *yfiLMN* deletion was complemented at *lacA* using the intergenic sequence between the terminator downstream of *yfiK* and the first coding nucleotide of *yfiL* as upstream sequence (143 bp). Lysis was observed in a strain lacking *yfiJK* (PDS0687), a strain with *yfiJK* ^+^ (PDS0688), and in strains with LDA^R^ alleles *yfiJ* ^A152E^ *K* (PDS0689) and *yfiJK* ^T83I^ (PDS0690). All cultures place *S*. Mg1 on the left and *B*. *subtilis* on the right. Cultures were photographed after 72 h co-incubation on MYM agar plates. Scale bar is 5 mm. These results were consistent across three replicates.
(TIFF)

S5 Fig Small colony formation requires *yfiJK*. We cultured eighteen wild type (PDS0066) and *ΔyfiJK* (PDS0554) colonies of *B*. *subtilis* with *S*. Mg1. Many small, potentially LDA^R^, colonies appeared in the region of lysis of wild type colonies. A few small colonies appeared in the zone of lysis of two *ΔyfiJK* colonies, but these did not grow similarly and lacked the morphological features of the *yfiJK* ^+^ small colonies. All cultures place *S*. Mg1 on the left and *B*. *subtilis* on the right. Colonies were photographed after 96 hours co-incubation on MYM agar plates. The scale bar is 5 mm.
(TIFF)

S6 Fig *yfiL* expression is increased in a LDA^R^ mutant. qRT-PCR was used to quantify expression of *yfiL* in strains with *yfiJK* ^+^ (PDS0627) or *yfiJ* ^A152E^ *K* (PDS0685). Expression was normalized relative to *gyrB*. The fold difference relative to expression in the *yfiJK* ^+^ strain is reported. The error bars represent the standard deviation of the fold difference.
(TIFF)

S1 Table ^13^C chemical shifts for linearmycin B. Spectra were collected in DMSO-d_6_ on a Bruker Avance 500 MHz spectrometer equipped with a cryoprobe. Chemical shifts (δ) are reported in ppm. Designated carbons are listed. The carbon number (C-No.) is with reference to the numbering in S1 Fig.
(PDF)

S2 Table Mutations in spontaneous LDA^R^ mutants not related to *yfiJK*. All numbering is with respect to the first amino acid or the first nucleotide of the start codon. *Mutations identified in the same spontaneous mutant. ^†^Mutations identified in a transposon-mutagenized strain.
(PDF)

S3 Table Strains of *Bacillus subtilis* used in this study. (PDF)

S4 Table Primers used in this study. (PDF)

S1 Methods Transposon mutagenesis. (PDF)

S1 Movie Time lapse movie of lysis and degradation of *B*. *subtilis* by *S*. Mg1. We co-cultured *S*. Mg1 and *B*. *subtilis Δpks* (PDS0067) on MYM agar and observed lysis and degradation of the *B*. *subtilis* colony. We cultured the strains at 30°C for 24 h before taking time lapse images at ambient temperature. We took images at 10 min intervals. See Fig 1A for scale bar.
(AVI)
