# Correction: Escape from Lethal Bacterial Competition through Coupled Activation of Antibiotic Resistance and a Mobilized Subpopulation

**DOI:** 10.1371/journal.pgen.1005807

**Published:** 2016-01-11

**Authors:** Reed M. Stubbendieck, Paul D. Straight

There is an error in the bond configuration for the linearmycin B structure depicted in Fig 1D. Please view the correct figure here.

**Fig 1:**
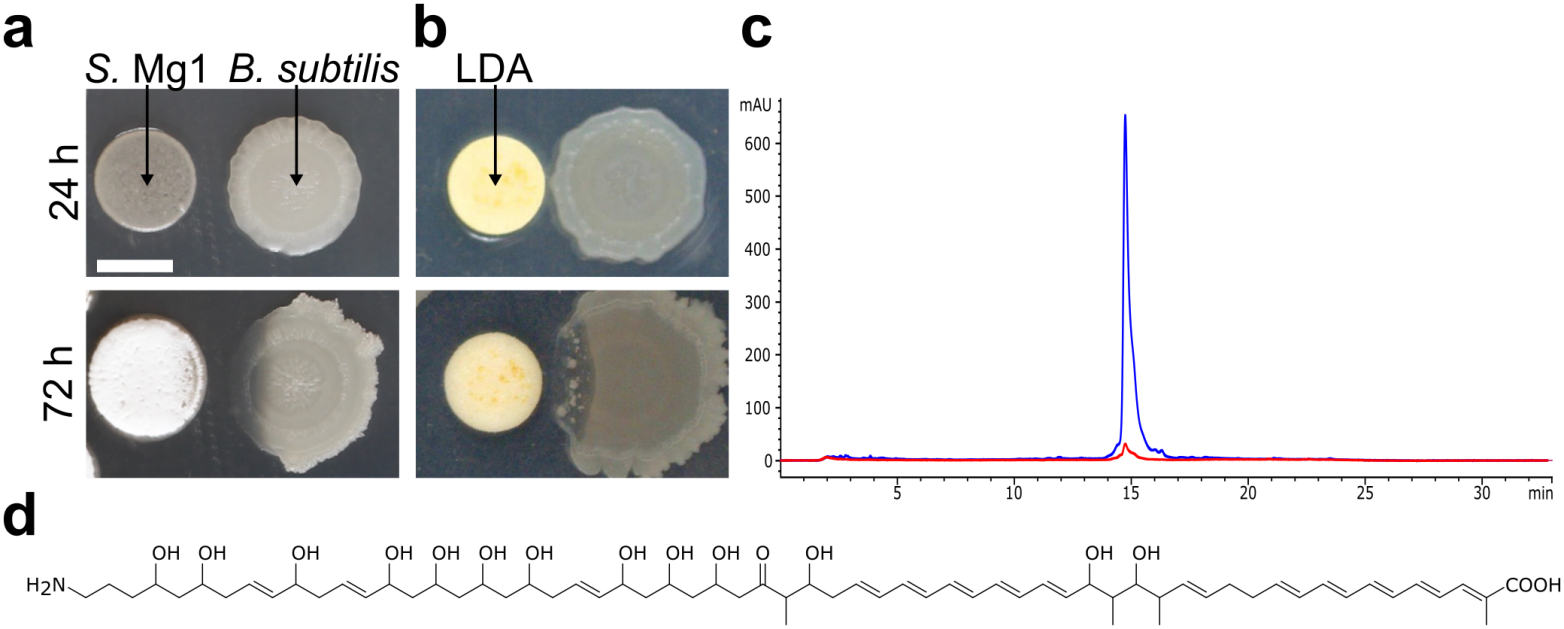
Identification of linearmycin B as the causative agent of LDA. (a) When co-cultured on MYM agar, *S*. Mg1 (left) releases molecule(s) that cause cellular lysis and colony degradation of *B*. *subtilis* (PDS0067) (right) at a distance. (b) We cultured *B*. *subtilis* (PDS0067) (right) alone on MYM7 agar for 24 h before adding isolated LDA onto a filter paper disc (left) adjacent to the colony, which subsequently lysed over 48 h similarly to co-culture with *S*. Mg1. (c) HPLC trace of the isolated LDA. The peak is detected by UV absorbance at 333 nm (blue). The background is shown by the 254 nm absorbing trace (red). (d) The structure of linearmycin B. Scale bar is 5 mm.

The images for Figs 6 and 7 are incorrectly switched. The image that appears as Fig 6 should be Fig 7, and the image that appears as Fig 7 should be Fig 6. The figure captions appear in the correct order. In S3 Fig the bottom colony is missing the *ΔlytF* label. Please view the correct figures here.

**Fig 6:**
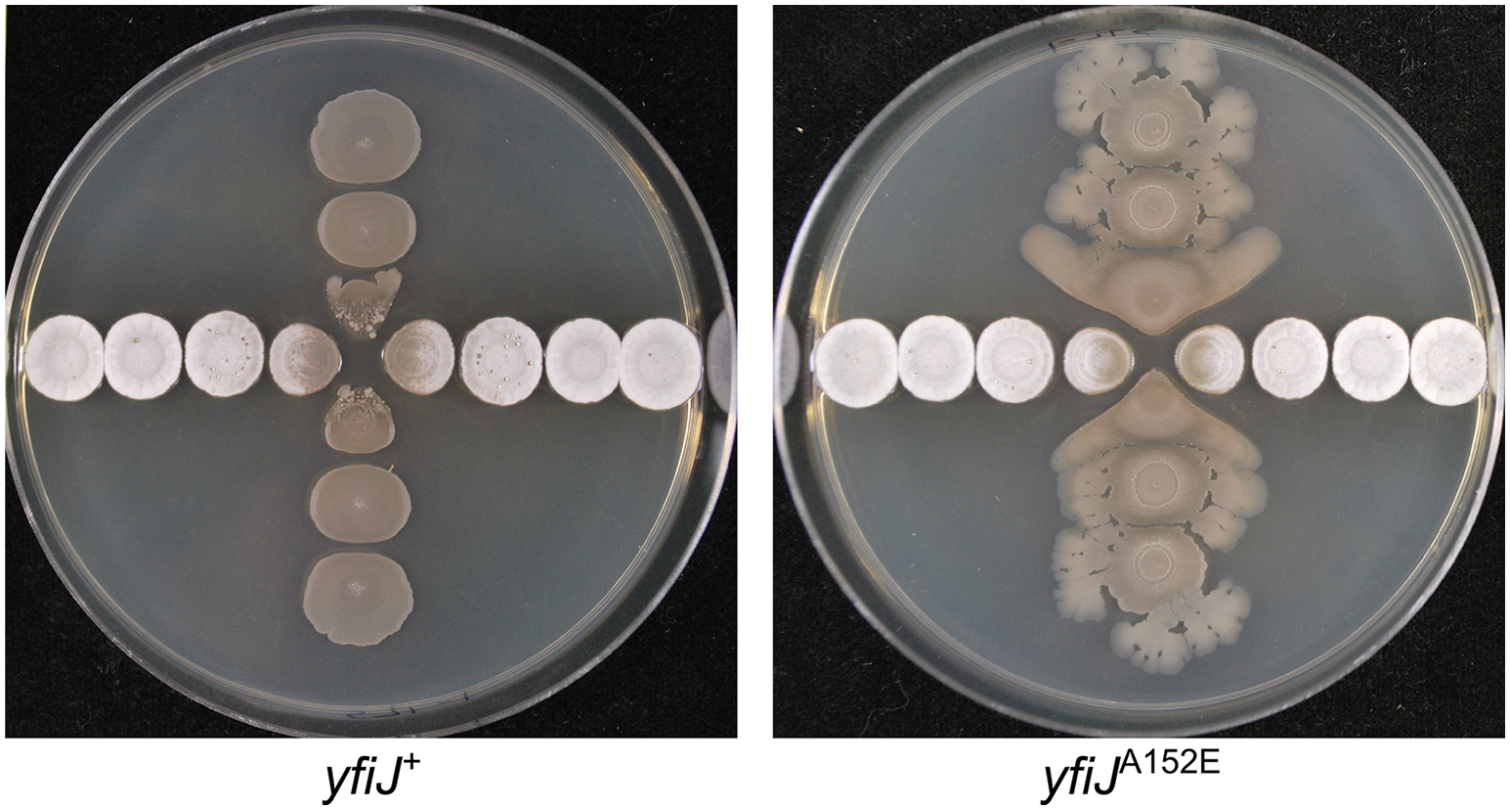
LDA^R^ mutants display a visible response to *S*. Mg1 in addition to inherent colony phenotypes. Wild type and LDA^R^ mutants were spotted in a perpendicular pattern, cross-wise to each other- *B*. *subtilis* (vertical) and *S*. Mg1 (horizontal). (Left) Strains of *B*. *subtilis* with *yfiJ*^+^ (PDS0571) have flat, immotile colonies. Proximal to *S*. Mg1, the colonies are lysed and degraded. (Right) Strains of *B*. *subtilis* with the LDA^R^ allele *yfiJ*^A152E^ (PDS0572) develop heterogeneous colonies, having wrinkled surfaces and motile outgrowths. Notably, the colonies of *yfiJ*^A152E^ *B*. *subtilis* near *S*. Mg1 have a distinctive spreading morphology. Plates were photographed after 96 hours co-incubation on MYM + 1.5% agar. Plates represent duplicate experiments.

**Fig 7:**
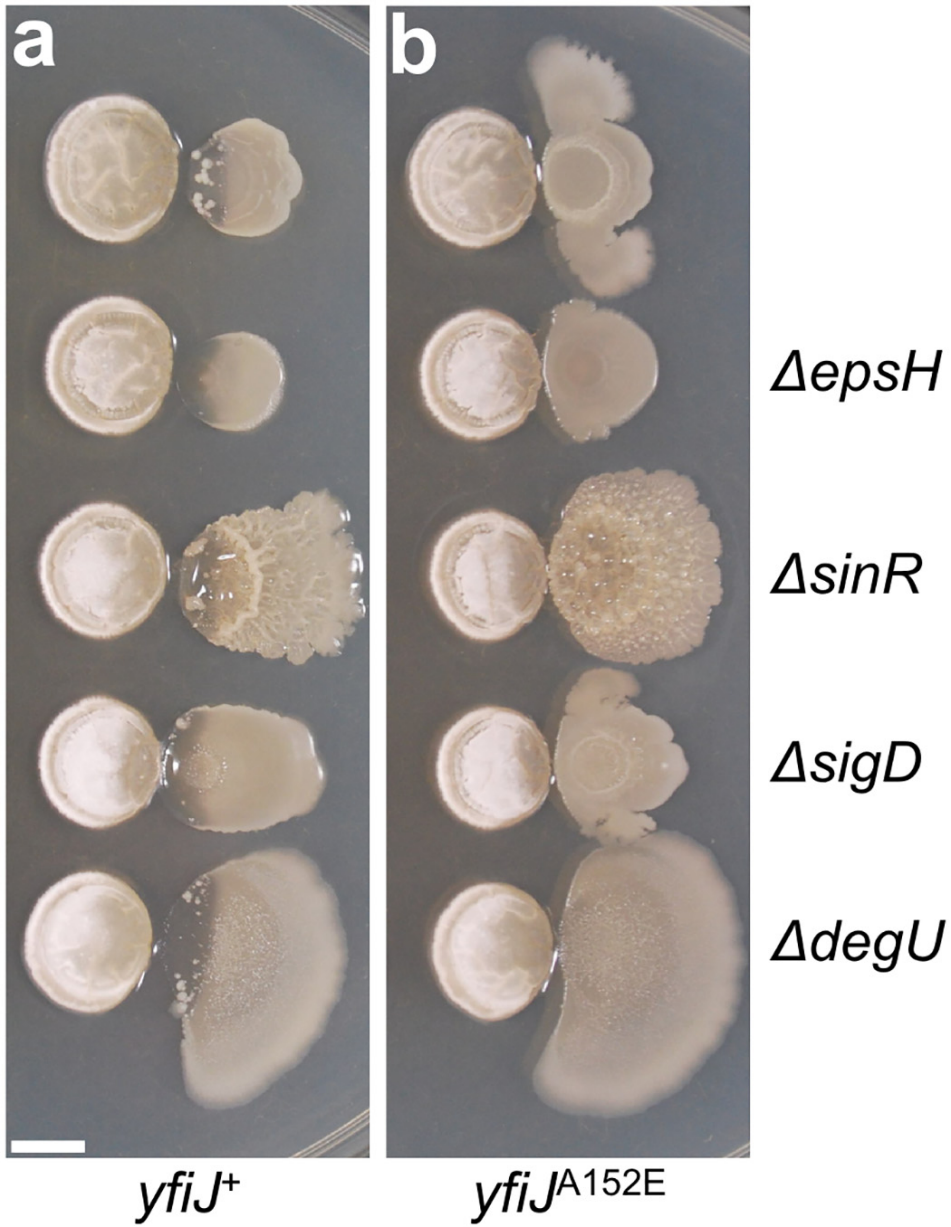
Colony morphology and LDA resistance are separable phenotypes. Genes involved in biofilm formation (*epsH*, *sinR*, and *degU*) and motility (*sigD*) were deleted from strains with either (a) *yfiJ*^+^ (PDS0571) or (b) the LDA^R^ allele *yfiJ*^A152E^ (PDS0572). In all cases, the biofilm and motility mutant strains were sensitive to lysis with wild-type *yfiJ* and were resistant to lysis with *yfiJ*^A152E^. All cultures place *S*. Mg1 on the left and *B*. *subtilis* on the right. Colonies were photographed after 72 hours co-incubation with *S*. Mg1 on MYM medium. Images are representative of triplicate samples. Scale bar is 5 mm.

There is an error in the NCBI BioProject Accession number in the Data Availability Statement and in the RNA-seq section of the Materials and Methods. The correct Accession number is PRJNA295934.

## Supporting Information

S3 Fig Genes predicted to be repressed by YfiK are not responsible for LDA. Spore-killing factor (SKF) and autolysis were predicted to be regulated by YfiK. Strains of *B*. *subtilis* (right) with deletions in genes responsible for SKF biosynthesis (*ΔskfA-H*) (DL598), an autolysin inhibitor (*ΔiseA*) (PDS0785), deletions in the major autolysin regulator σ^D^ (*ΔsigD*) (DS323), and deletions in three major autolysins (*ΔlytABC*, *ΔlytD*, *ΔlytF*) (DS2483) were tested for resistance to LDA in co-culture with *S*. Mg1 (left). All strains lysed similarly to wild type (PDS0066). Cultures were photographed after 72 h co-incubation on MYM agar plates. Scale bar is 5 mm. These results were consistent across six replicates.
(TIFF)
