# Supplementary figures and images for: Escape from Lethal Bacterial Competition through Coupled Activation of Antibiotic Resistance and a Mobilized Subpopulation
Source: PLoS Genet. 2015 Dec 8;11(12):e1005722. doi: 10.1371/journal.pgen.1005722 (PMC4672918; doi:10.1371/journal.pgen.1005722)

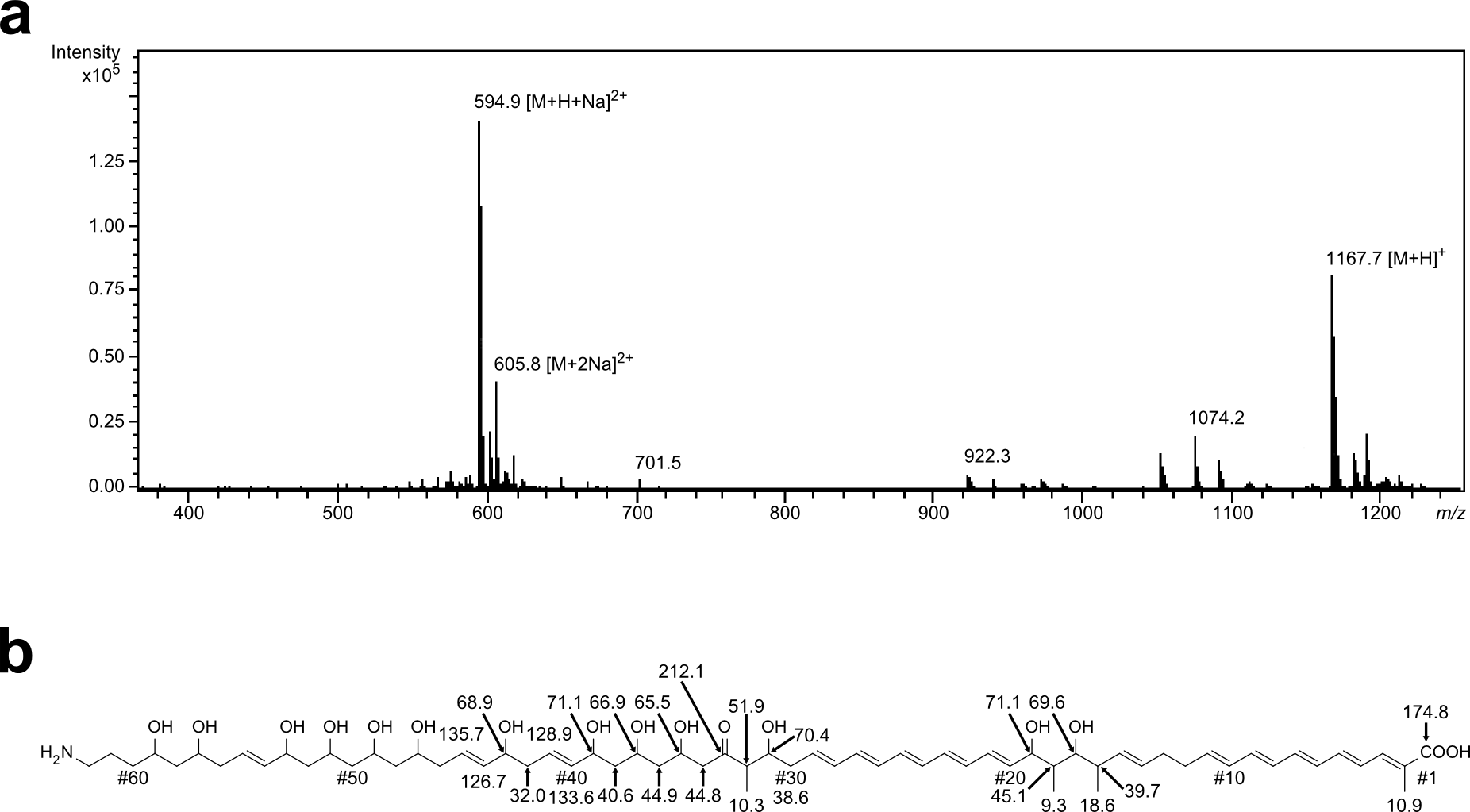

Supplement: S1 Fig — (a) Mass spectrum of the isolated peak with lytic activity against B. subtilis. The prominent masses detected in the experiment match those of linearmycin B, m/z 594.9 [M+H+Na]2+, 605.8 [M+2Na]2+ and 1167.7 [M+H]+ (b) Structure of linearmycin B with 13C NMR chemical shift assignments obtained in DMSO-d6. Carbons are numbered linearly starting with the carbonyl carbon of the carboxylic acid group. (TIFF) [file pgen.1005722.s001.tiff]

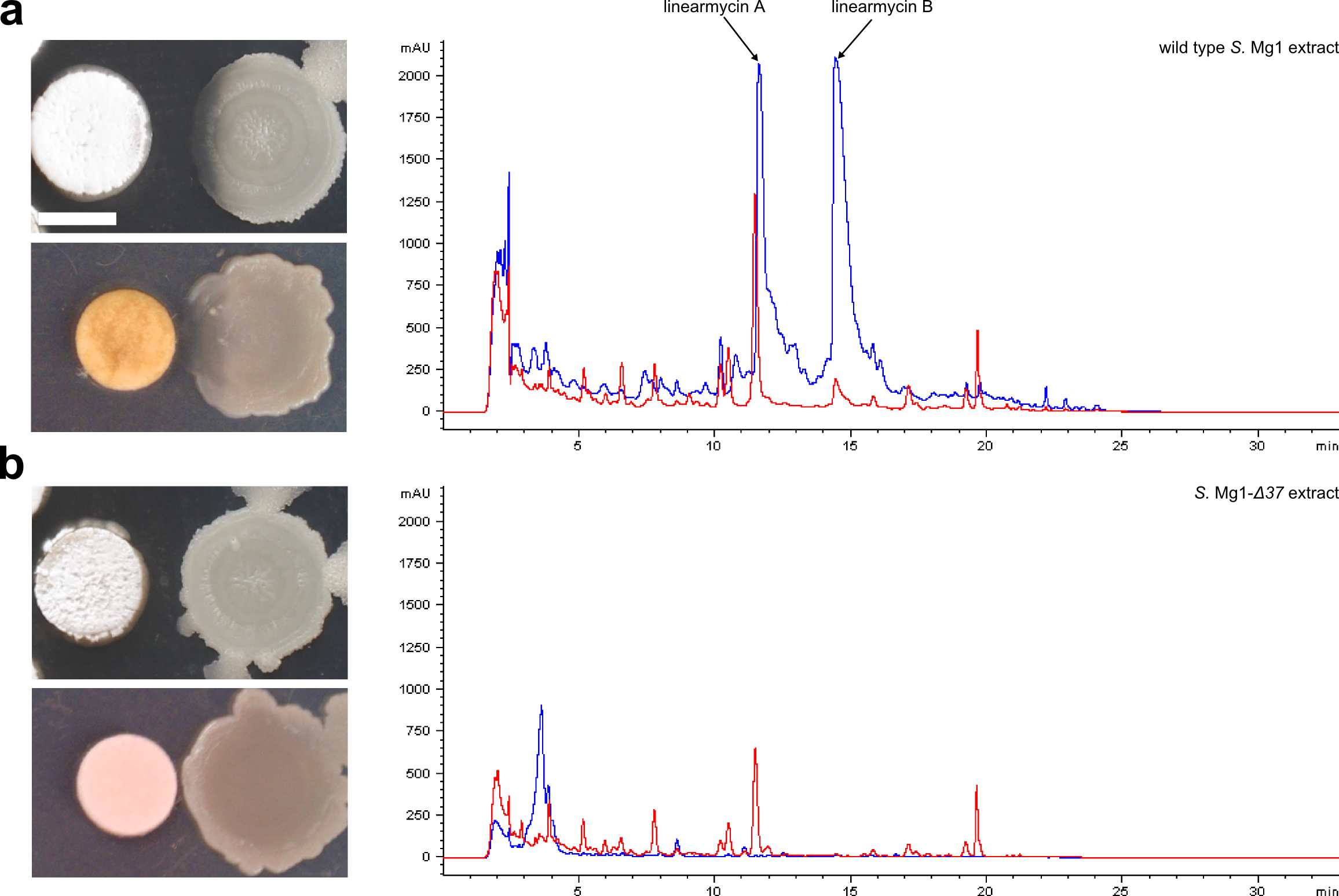

Supplement: S2 Fig — The S. Mg1 wild type strain (a) and a strain with a chromosome arm deletion (Δ37) that includes the linearmycin biosynthetic gene cluster (b) were co-cultured with B. subtilis Δpks (PDS0067) (top left panels). Bacillus subtilis is not lysed by S. Mg1 Δ37. Extracts from each streptomycete were spotted on filter paper discs adjacent to a B. subtilis Δpks colony (lower left panels). The B. subtilis colony challenged with the S. Mg1 Δ37 extract was not lysed. The extracts were analyzed by HPLC (right panels). Linearmycins are detected by UV absorbance at 333 nm (blue) while the background is shown by the 254 nm absorbing trace (red). The predominant difference in the extracts is the presence or absence of linearmycins A and B. Linearmycin A (m/z 1140) and B (m/z 1166) identities were confirmed by mass spectrometry. Colonies were photographed after 72 h of co-incubation or after 48 h exposure to extract. Scale bar is 5 mm. (TIFF) [file pgen.1005722.s002.tiff]

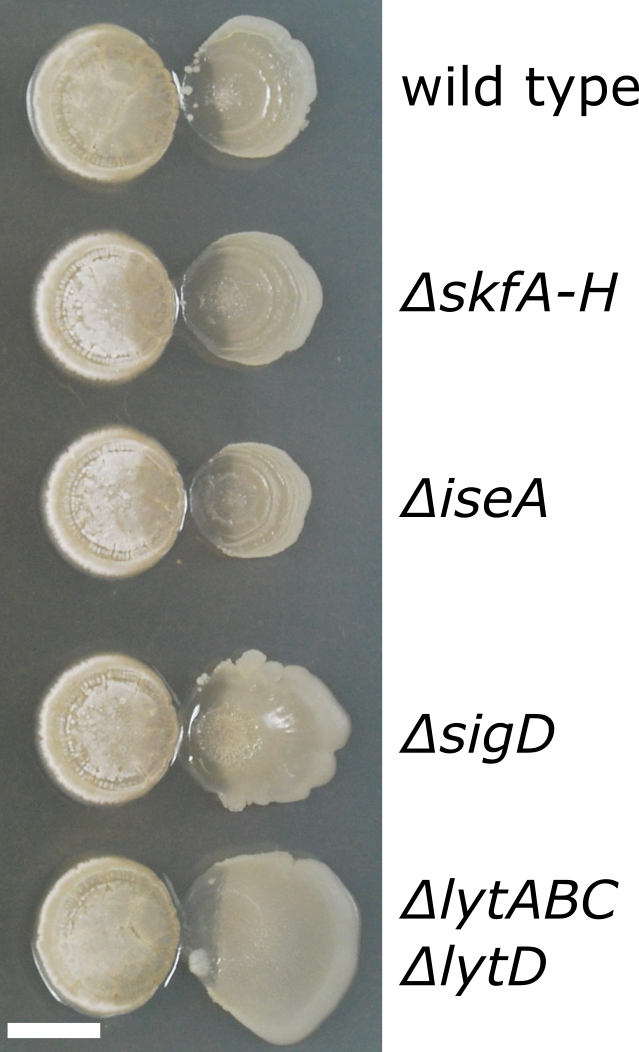

Supplement: S3 Fig — Spore-killing factor (SKF) and autolysis were predicted to be regulated by YfiK. Strains of B. subtilis (right) with deletions in genes responsible for SKF biosynthesis (ΔskfA-H) (DL598), an autolysin inhibitor (ΔiseA) (PDS0785), deletions in the major autolysin regulator σD (ΔsigD) (DS323), and deletions in three major autolysins (ΔlytABC, ΔlytD, ΔlytF) (DS2483) were tested for resistance to LDA in co-culture with S. Mg1 (left). All strains lysed similarly to wild type (PDS0066). Cultures were photographed after 72 h co-incubation on MYM agar plates. Scale bar is 5 mm. These results were consistent across six replicates. (TIFF) [file pgen.1005722.s003.tiff]

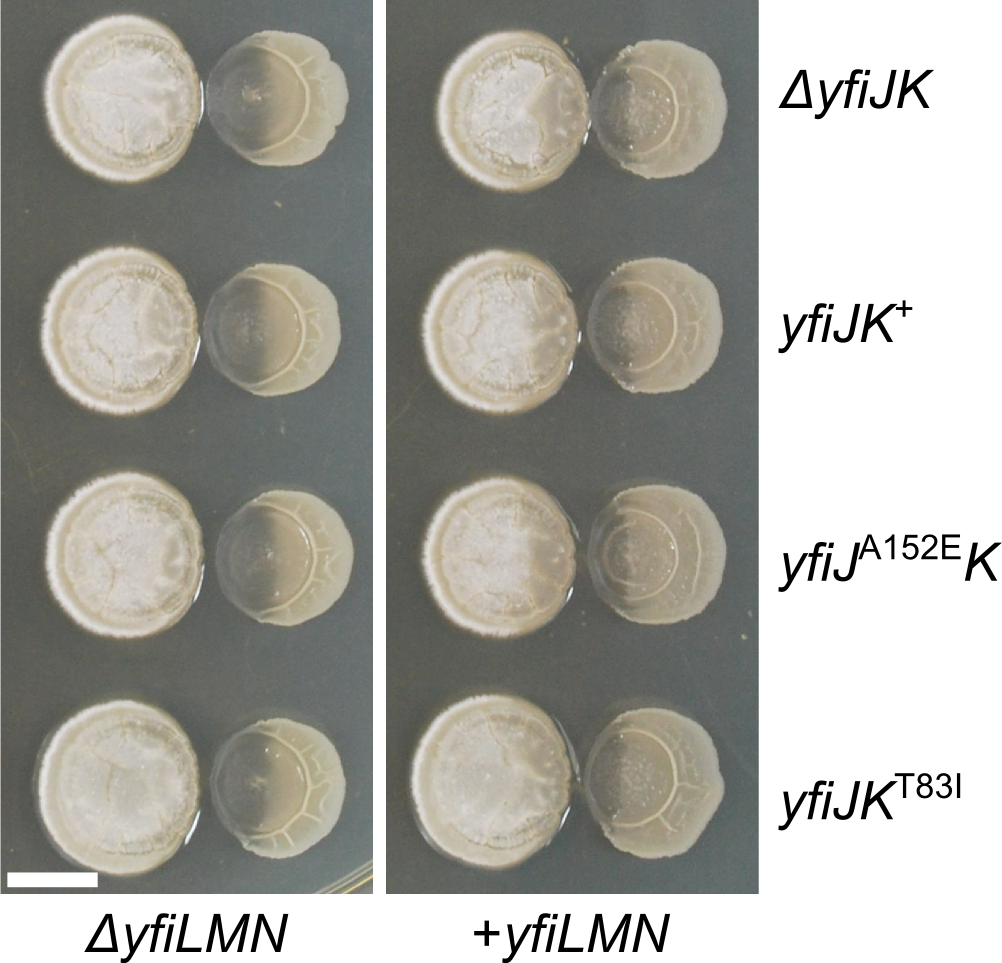

Supplement: S4 Fig — The yfiLMN deletion was complemented at lacA using the intergenic sequence between the terminator downstream of yfiK and the first coding nucleotide of yfiL as upstream sequence (143 bp). Lysis was observed in a strain lacking yfiJK (PDS0687), a strain with yfiJK + (PDS0688), and in strains with LDAR alleles yfiJ A152E K (PDS0689) and yfiJK T83I (PDS0690). All cultures place S. Mg1 on the left and B. subtilis on the right. Cultures were photographed after 72 h co-incubation on MYM agar plates. Scale bar is 5 mm. These results were consistent across three replicates. (TIFF) [file pgen.1005722.s004.tiff]

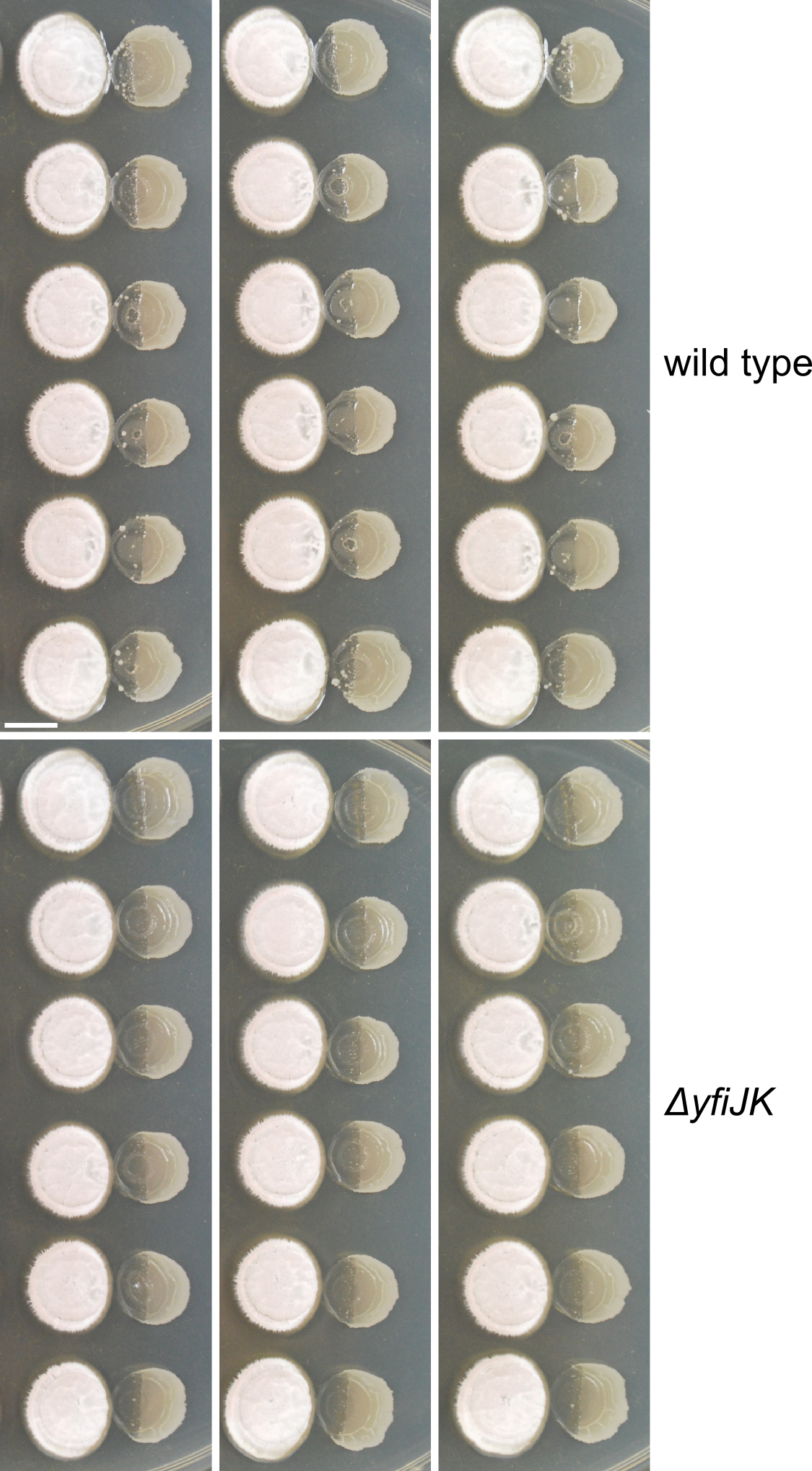

Supplement: S5 Fig — We cultured eighteen wild type (PDS0066) and ΔyfiJK (PDS0554) colonies of B. subtilis with S. Mg1. Many small, potentially LDAR, colonies appeared in the region of lysis of wild type colonies. A few small colonies appeared in the zone of lysis of two ΔyfiJK colonies, but these did not grow similarly and lacked the morphological features of the yfiJK + small colonies. All cultures place S. Mg1 on the left and B. subtilis on the right. Colonies were photographed after 96 hours co-incubation on MYM agar plates. The scale bar is 5 mm. (TIFF) [file pgen.1005722.s005.tiff]

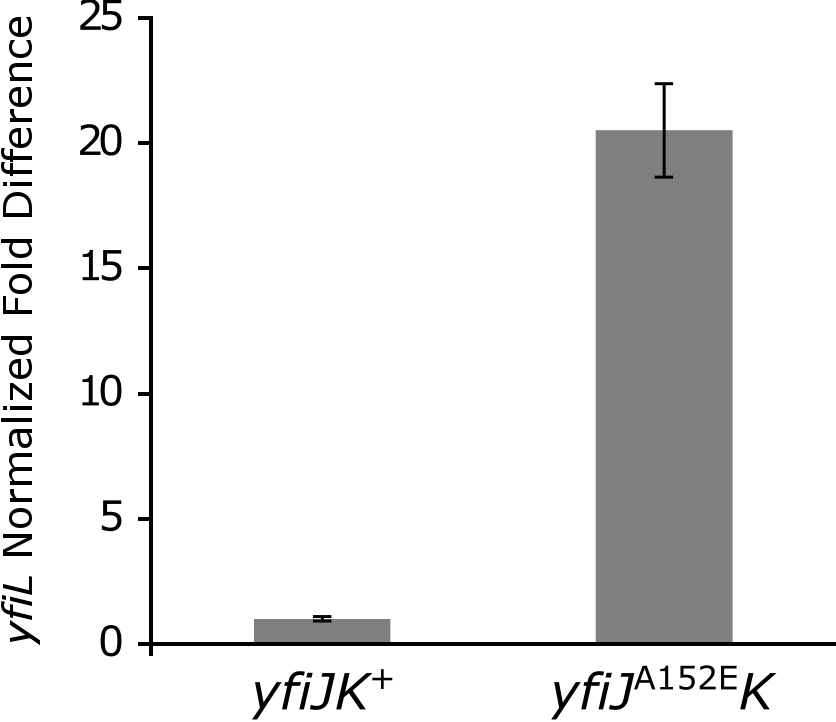

Supplement: S6 Fig — qRT-PCR was used to quantify expression of yfiL in strains with yfiJK + (PDS0627) or yfiJ A152E K (PDS0685). Expression was normalized relative to gyrB. The fold difference relative to expression in the yfiJK + strain is reported. The error bars represent the standard deviation of the fold difference. (TIFF) [file pgen.1005722.s006.tiff]
