# Supplementary material for: Escape from Lethal Bacterial Competition through Coupled Activation of Antibiotic Resistance and a Mobilized Subpopulation
Source: PLoS Genet. 2015 Dec 8;11(12):e1005722. doi: 10.1371/journal.pgen.1005722 (PMC4672918; doi:10.1371/journal.pgen.1005722)
Supplement: S1 Table — Spectra were collected in DMSO-d6 on a Bruker Avance 500 MHz spectrometer equipped with a cryoprobe. Chemical shifts (δ) are reported in ppm. Designated carbons are listed. The carbon number (C-No.) is with reference to the numbering in S1 Fig. (PDF) [file pgen.1005722.s007.pdf]

Supplemental Table S1. ^{13}C Chemical Shifts for Linearmycin B.

C-No.	δ_C	C-No.	δ_C	C-No.	δ_C	C-No.	δ_C	C-No.	δ_C
33	212.07		132.40		75.13		45.83		24.27
1	174.78		132.09	39	71.12	18	45.11		24.18
	138.52		131.75	19	71.09	36	44.98	16-Me	18.55
	136.85		131.29	31	70.41	34	44.88	2-Me	10.91
45	135.69		131.24	17	69.58	38	40.64	32-Me	10.26
	135.32		131.10	43	68.90	16	39.65	18-Me	9.25
	135.25		130.02		68.76	30	38.58		
	134.97		128.98		68.48		34.45		
40	133.62	41	128.91		67.75		32.68		
	133.56		128.75	37	66.86	42	31.91		
	133.22	44	126.72	35	65.49		31.39		
	132.93		126.65		63.83		29.91		
	132.86		126.38		63.65		29.13		
	132.80		125.06	32	51.86		28.82		
	132.74		125.01		48.70		28.47		

Spectra were collected in DMSO-d6 on a Bruker Avance 500 MHz spectrometer equipped with a cryoprobe. Chemical shifts (δ) are reported in ppm with reference to the residual solvent peak. Designated carbons are listed. The carbon number (C-No.) is with reference to the numbering in Fig S1b.
