# Supplementary material for: Escape from Lethal Bacterial Competition through Coupled Activation of Antibiotic Resistance and a Mobilized Subpopulation
Source: PLoS Genet. 2015 Dec 8;11(12):e1005722. doi: 10.1371/journal.pgen.1005722 (PMC4672918; doi:10.1371/journal.pgen.1005722)
Supplement: S2 Table — All numbering is with respect to the first amino acid or the first nucleotide of the start codon. *Mutations identified in the same spontaneous mutant. †Mutations identified in a transposon-mutagenized strain. (PDF) [file pgen.1005722.s008.pdf]

Supplemental Table S2. Mutations in spontaneous LDA^{R} mutants not related to *yfiJK*.

Gene	Nucleotide change	Effect	Annotation
fosB	G57T	none	bacillithiol-S-transferase, fosfomycin resistance protein
rnc	C299T	P100L	RNase III
nrdEB[*]	A622G	K208E	SPβ phage ribonucleoside-diphosphate reductase, alpha subunit
yodT[*]	C582T	none	putative aminovalerate aminotransferase
yozT[*]	A66G	none	unknown
ypzE[*]	A124G	K42E	unknown
nadD[†]	T392A	V131E	nicotinate-nucleotide adenylyltransferase
recJ[†]	C499T	A150V	single-strand DNA-specific exonuclease
tpx[†]	A209G	E70G	putative peroxiredoxin
yhgB[†]	32_33insG	frame shift	unknown
yopC[†]	*yopC*ΩTnYLB-1	disruption	unknown membrane protein

All numbering is with respect to the first amino acid or the first nucleotide of the start codon. [*]Mutations identified in the same spontaneous mutant. [†]Mutations identified in a transposon-mutagenized strain.
