# Supplementary material for: Escape from Lethal Bacterial Competition through Coupled Activation of Antibiotic Resistance and a Mobilized Subpopulation
Source: PLoS Genet. 2015 Dec 8;11(12):e1005722. doi: 10.1371/journal.pgen.1005722 (PMC4672918; doi:10.1371/journal.pgen.1005722)
Supplement: S3 Table — (PDF) [file pgen.1005722.s009.pdf]

Supplemental Table S3. Strains of *Bacillus subtilis* used in this study.

Strain	Genotype	Source
PDS0066	NCIB3610 undomesticated wild type strain	Laboratory collection
PDS0067	NCIB3610 *ΔpksX::spc*	Laboratory collection
PDS0121	NCIB3610 pMarA	Laboratory collection
PDS0312	PY79 wild type	Laboratory collection
PDS0540	PY79 pDR244	Laboratory collection
PDS0546	PY79 *ΔyfiJK::kn*	This study
PDS0547	NCIB3610 *ΔyfiJ::mls*	This study
PDS0548	NCIB3610 *ΔyopC::mls*	This study
PDS0553	NCIB3610 *ΔyfiK::mls*	This study
PDS0554	NCIB3610 *ΔyfiJK::kn*	This study
PDS0555	NCIB3610 *ΔyfiJ*	This study
PDS0556	NCIB3610 *ΔyfiK*	This study
PDS0559	168 *ΔyfiJ*	This study
PDS0562	168 *ΔyfiJ lacA::yfiJ* (*mls*)	This study
PDS0563	168 *ΔyfiJ lacA::yfiJ*^{A152E} (*mls*)	This study
PDS0564	168 *ΔyfiJ lacA::yfiJ*^{T164M} (*mls*)	This study
PDS0565	168 *ΔyfiJ lacA::yfiJ*^{H167Y} (*mls*)	This study
PDS0566	168 *ΔyfiJ lacA::yfiJ*^{A88V} (*mls*)	This study
PDS0567	168 *ΔyfiJ lacA::yfiJ*^{H201N} (*mls*)	This study
PDS0571	NCIB3610 *ΔyfiJ lacA::yfiJ* (*mls*)	This study
PDS0572	NCIB3610 *ΔyfiJ lacA::yfiJ*^{A152E} (*mls*)	This study
PDS0573	NCIB3610 *ΔyfiJ lacA::yfiJ*^{T164M} (*mls*)	This study
PDS0574	NCIB3610 *ΔyfiJ lacA::yfiJ*^{H167Y} (*mls*)	This study
PDS0575	NCIB3610 *ΔyfiJ lacA::yfiJ*^{A88V} (*mls*)	This study
PDS0576	NCIB3610 *ΔyfiJ lacA::yfiJ*^{H201N} (*mls*)	This study
PDS0594	168 *ΔyfiJ lacA::yfiJ*$^{A152E, H201N}$ (*mls*)	This study
PDS0604	NCIB3610 *ΔyfiJ lacA::yfiJ*$^{A152E, H201N}$ (*mls*)	This study
PDS0608	NCIB3610 *ΔyfiJ ΔepsH::kn lacA::yfiJ*^{A152E} (*mls*)	This study
PDS0623	PY79 *ΔyfiJK::kn amyE::yfiJK* (*spc*)	This study
PDS0624	PY79 *ΔyfiJK::kn amyE::yfiJK*^{T83I} (*spc*)	This study
PDS0625	PY79 *ΔyfiJK::kn amyE::yfiJK*^{D54A} (*spc*)	This study
PDS0626	PY79 *ΔyfiJK::kn amyE::yfiJK*$^{D54A, T83I}$ (*spc*)	This study
PDS0627	NCIB3610 *ΔyfiJK::kn amyE::yfiJK (spc)*	This study
PDS0628	NCIB3610 *ΔyfiJK::kn amyE::yfiJK*^{T83I} *(spc)*	This study
PDS0629	NCIB3610 *ΔyfiJK::kn amyE::yfiJK*^{D54A} *(spc)*	This study
PDS0630	NCIB3610 *ΔyfiJK::kn amyE::yfiJK*$^{D54A, T83I}$ *(spc)*	This study
PDS0652	PY79 *ΔyfiJKLMN::kn*	This study
PDS0653	NCIB3610 *ΔyfiJKLMN::kn*	This study
PDS0658	NCIB3610 *ΔyfiJKLMN::kn amyE::yfiJK (spc)*	This study
PDS0660	NCIB3610 *ΔyfiJKLMN::kn amyE::yfiJK*^{T83I} *(spc)*	This study
PDS0685	NCIB3610 *ΔyfiJK::kn amyE::*yfiJ^{A152E}*K (spc)*	This study
PDS0686	NCIB3610 *ΔyfiJKLMN::kn amyE::yfiJ*^{A152E}*K (spc)*	This study
PDS0687	NCIB3610 *ΔyfiJKLMN::kn lacA::yfiLMN (mls)*	This study
PDS0688	NCIB3610 *ΔyfiJKLMN::kn amyE::yfiJK (spc) lacA::yfiLMN (mls)*	This study
PDS0689	NCIB3610 *ΔyfiJKLMN::kn amyE::yfiJ*^{A152E}*K (spc) lacA::yfiLMN (mls)*	This study
PDS0690	NCIB3610 *ΔyfiJKLMN::kn amyE::yfiJK*^{T83I} *(spc) lacA::yfiLMN (mls)*	This study
PDS0691	PY79 *ΔyfiJKLMN::kn lacA::yfiLMN (mls)*	This study
PDS0717	PY79 *ΔyfiJKLMN::kn yhdG::*P$_{spac(c)}$*yfiLMN (cm)*	This study
PDS0718	NCIB3610 *ΔyfiJKLMN::kn yhdG::*P$_{spac(c)}$*yfiLMN (cm)*	This study
PDS0719	NCIB3610 *ΔyfiJKLMN::kn amyE::yfiJK (spc) yhdG::*P$_{spac(c)}$*yfiLMN (cm)*	This study
PDS0720	NCIB3610 *ΔyfiJKLMN::kn amyE::yfiJ*^{152E}*K (spc) yhdG::*P$_{spac(c)}$*yfiLMN (cm)*	This study
PDS0721	NCIB3610 *ΔyfiJKLMN::kn amyE::yfiJK*^{T83I} *(spc) yhdG::*P$_{spac(c)}$*yfiLMN (cm)*	This study
PDS0731	NCIB3610 *ΔyfiJ ΔepsH::kn lacA::yfiJ (mls)*	This study
PDS0732	NCIB3610 *ΔyfiJ ΔsinR::spc lacA::yfiJ (mls)*	This study
PDS0733	NCIB3610 *ΔyfiJ ΔsigD::tet lacA::yfiJ (mls)*	This study
PDS0734	NCIB3610 *ΔyfiJ ΔdegU::tet lacA::yfiJ (mls)*	This study
PDS0735	NCIB3610 *ΔyfiJ ΔsinR::spc lacA::yfiJ*A^{152E} *(mls)*	This study
PDS0736	NCIB3610 *ΔyfiJ ΔsigD::tet lacA::yfiJ*A^{152E} *(mls)*	This study
PDS0737	NCIB3610 *ΔyfiJ ΔdegU::tet lacA::yfiJ*A^{152E} *(mls)*	This study
PDS0738	NCIB3610 *ΔyfiJ lacA::yfiJ*^{L254P} (*mls*)	This study
PDS0739	NCIB3610 *ΔyfiJK::kn amyE::yfiJ*^{A152E}*K*^{D54A} *(spc)*	This study
PDS0740	NCIB3610 *ΔyfiJK::kn amyE::yfiJ*^{H201N}*K*^{T83I} *(spc)*	This study
PDS0785	NCIB3610 *ΔiseA::mls*	This study
BKE18380	168 *ΔiseA::mls*	*Bacillus* Genetic Stock Center
BKE20940	168 *ΔyopC::mls*	*Bacillus* Genetic Stock Center
BKE08290	168 *ΔyfiJ::mls*	*Bacillus* Genetic Stock Center
BKE08300	168 *ΔyfiK::mls*	*Bacillus* Genetic Stock Center
DL598	NCIB3610 *ΔskfABCDEFGH::cm*	R. Kolter Laboratory
DS323	NCIB3610 *ΔsigD::tet*	D. Kearns Laboratory
DS2483	NCIB3610 *ΔlytABC::kn ΔlytD::mls ΔlytF::tet*	D. Kearns Laboratory
