# Supplementary material for: Escape from Lethal Bacterial Competition through Coupled Activation of Antibiotic Resistance and a Mobilized Subpopulation
Source: PLoS Genet. 2015 Dec 8;11(12):e1005722. doi: 10.1371/journal.pgen.1005722 (PMC4672918; doi:10.1371/journal.pgen.1005722)
Supplement: S4 Table — (PDF) [file pgen.1005722.s010.pdf]

Supplemental Table S4. Primers used in this study.

Primer	Sequence (5′ - 3′)
kn-Fwd	CAGCGAACCATTTGAGGTGATAGG
kn-Rev	CGATACAAATTCCTCGTAGGCGCTCGG
13: *yfiJ*-up-fwd	GGCAGGAAATCAAAGCGCTC
14: *yfiJ*-up-rev	GCGCCTACGAGGAATTTGTATCGCCATCAAGGTGAACATTCTCGT
15: *yfiK*-down-dwd	CCTATCACCTCAAATGGTTCGCTGAGAACCCAAGCCGCCATTTA
16: *yfiK*-down-rev	TTTCATTGCCGTCCCTCCTC
25: *yfiJ*-fwd(BamHI)	CCATGGATCCATTGATGCAGGGATCGAGGG
26: *yfiJ*-rev(EcoRI)	TCATGAATTCGATGCCAGCCCTTCTCTGAC
42: $yfiJ^{H201}$-fwd	GGCGCGTGAAATCAATGATACAGTGGGG
43: $yfiJ^{H201}$-rev	CCCCACTGTATCATTGATTTCACGCGCC
50: $yfiK^{D54A}$-fwd	GGATATCGTGTTAATGGCCATCCGCATGCCGGTTTC
51: $yfiK^{D54A}$-rev	GAAACCGGCATGCGGATGGCCATTAACACGATATCC
54: *yfiJK*-Fwd	GCGACCGGCGCTCAGGATCCATTGATGCAGGGATCGAGGG
59: pDR111-rev	CCCTCGATCCCTGCATCAATGGATCCTGAGCGCCGGTCGC
74: pDR111-fwd	TATGTTCTATCTGCCGCTACGAATTCCTGCAGCCCTGGCG
75: *yfiJK*-Rev	GTAGCGGCAGATAGAACATA
76: *yfiJ*-up-fwd	GGCCGCCCGCGGTAGGATCCTTGTAAGGCGGCGCTTGAAG
77: *yfiJ*-up-rev	CCTACGAGGAATTTGTATCGGCTCATCACTCCCGATACCC
78: *yfiN*-down-fwd	TCACCTCAAATGGTTCGCTGAAAACATCTGCCGTTTAGGC
79: *yfiN*-down-rev	CCCGGGGAGCTCATGAATTCACGACAGGATTATGTACTGACTC
112: $P_{pac(c)}$-fwd(EcoRI)	ATGCGAATTCTACACAGCCCAGTC
113: $P_{pac(c)}$-fwd(SpeI)	ATGCACTAGTAACCGGATTCCACACATTATGCCAC
118: pRMS1-fwd	ACTAGTAACCGGATTCCACA
119: pRMS1-rev	GGATCCCATACGGCAATAGT
120: *yfiLMN*-fwd	TGTGGAATCCGGTTACTAGTAGGAGTGAGACGACGTGCTG
121: *yfiLMN*-rev	ACTATTGCCGTATGGGATCCTTAGGCTCGGAGCGCTTTCA
q1: *yfiL*_qPCR_fwd	AAGCGTTTCTTGTGGCGATC
q2: *yfiL*_qPCR_rev	TGATGAGCCGCAGAAATGTC
gyrB qPCR-fwd	GGGCAACTCAGAAGCACGGACG
gyrB qPCR-rev	GCCATTCTTGCTCTTGCCGCC
