# Supplementary material for: Escape from Lethal Bacterial Competition through Coupled Activation of Antibiotic Resistance and a Mobilized Subpopulation
Source: PLoS Genet. 2015 Dec 8;11(12):e1005722. doi: 10.1371/journal.pgen.1005722 (PMC4672918; doi:10.1371/journal.pgen.1005722)
Supplement: S1 Methods — (PDF) [file pgen.1005722.s011.pdf]

Transposon mutagenesis. We used a strain of *Bacillus subtilis* harboring the pMarA plasmid transposon mutagenesis system [1] (PDS0121) to identify genes that may cause lysis under linearmycin-induced stress. pMarA is a single copy plasmid that contains a *Himar1* transposase gene under control of the housekeeping sigma factor σ^A. Transposition occurs during growth and each cell should undergo a single transposition event. We diluted cultures of PDS0121 that were grown overnight at 22 °C to OD_{600} = 0.05 in 5 mL of LB with kanamycin (5 µg/mL). When the OD_{600} reached 0.3-0.4 we raised the temperature from 22 °C to 42 °C, which restricts replication of pMarA. When the culture reached OD_{600} = 1 (~10^9 cells/mL) we added 10 µL of LDA-containing and returned cultures to 42 °C. At this point the culture represented a library of transposon-insertion mutants. After ~3 hours of incubation, the OD_{600} decreased ≥ 10-fold upon cell lysis. We plated the surviving cells on LB containing LDA extract. Following incubation of the plates, we isolated ~200 survivors, which we subsequently passaged on LB without LDA extract. We then tested the passaged isolates for LDA resistance in culture with *S*. Mg1. Only a single stable LDA^R mutant passed through the selection. We mapped the transposon insertion to the *yopC* gene, which encodes a predicted membrane protein within the Spβ prophage sequence [2]. A *ΔyopC* strain was not LDA resistant in culture with *S.* Mg1. We backcrossed the mutant strain to wild type and found the transposon-associated marker was unlinked to LDA resistance. To locate the LDA^R-conferring allele, we sequenced this strain. The sequence revealed an additional point mutation in *yfiJ* (L254P substitution), which we confirmed for resistance to LDA (Table 1).

References

1. Breton Y Le, Mohapatra NP, Haldenwang WG. In Vivo Random Mutagenesis of Bacillus subtilis by Use of TnYLB-1, a mariner-Based Transposon. Appl Environ Microbiol. 2006;72: 327–333. doi:10.1128/AEM.72.1.327

2. Lazarevic V, Düsterhöft A, Soldo B, Hilbert H, Mauël C, Karamata D. Nucleotide sequence of the Bacillus subtilis temperate bacteriophage SPβc2. Microbiology. 1999;145 (Pt 5): 1055–67.
