# Supplementary figures and images for: Correction: Escape from Lethal Bacterial Competition through Coupled Activation of Antibiotic Resistance and a Mobilized Subpopulation
Source: PLoS Genet. 2016 Jan 11;12(1):e1005807. doi: 10.1371/journal.pgen.1005807 (PMC4709098; doi:10.1371/journal.pgen.1005807)

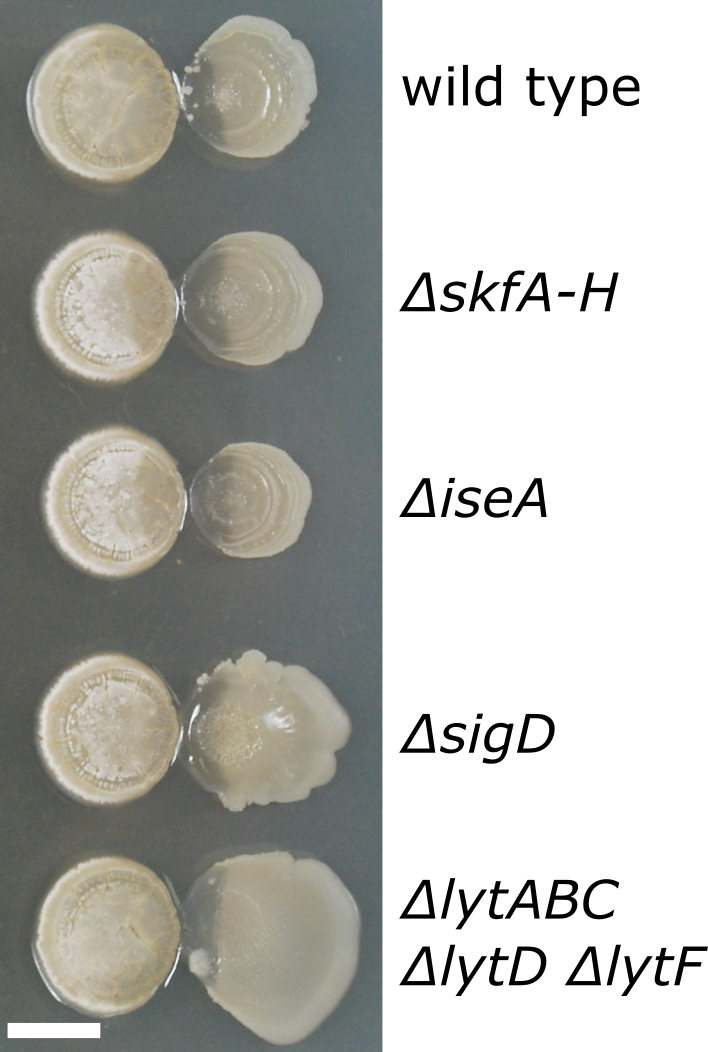

Supplement: S3 Fig — Spore-killing factor (SKF) and autolysis were predicted to be regulated by YfiK. Strains of B. subtilis (right) with deletions in genes responsible for SKF biosynthesis (ΔskfA-H) (DL598), an autolysin inhibitor (ΔiseA) (PDS0785), deletions in the major autolysin regulator σD (ΔsigD) (DS323), and deletions in three major autolysins (ΔlytABC, ΔlytD, ΔlytF) (DS2483) were tested for resistance to LDA in co-culture with S. Mg1 (left). All strains lysed similarly to wild type (PDS0066). Cultures were photographed after 72 h co-incubation on MYM agar plates. Scale bar is 5 mm. These results were consistent across six replicates. (TIFF) [file pgen.1005807.s001.tiff]
